# Exploring Female Students’ Experiences of ADHD and its Impact on Social, Academic, and Psychological Functioning

**DOI:** 10.1177/10870547231168432

**Published:** 2023-04-25

**Authors:** Eden Morley, Aimee Tyrrell

**Affiliations:** 1 University of Nottingham, Nottingham, UK

**Keywords:** ADD/ADHD, females, gender bias, ADHD-associated problems

## Abstract

**Objective:**

The aim of research is to provide greater understanding of ADHD in adult females by exploring first-hand experiences of female university students with ADHD in the UK, and the impact of such experiences on social, academic, and psychological functioning.

**Methods:**

Semi-structured interviews were conducted with eight adult women attending university in the UK—all clinically diagnosed with ADHD during adulthood.

**Results:**

Participants experiences were rich and insightful, identifying that many women with ADHD experience stigmatization and social discrimination, amongst other social, academic, and psychological difficulties.

**Conclusion:**

Overall, the research identifies the pressing need for greater understanding and appreciation of ADHD in females, particularly amongst health professionals.

## Introduction

Attention-deficit/hyperactivity disorder (ADHD) is a common neurodevelopmental disorder characterized by a persistent pattern of inattention and/or hyperactivity-impulsivity which interferes with daily functioning (American Psychiatric Association, 2013). There are three subtypes of ADHD; predominantly inattentive, predominantly hyperactive-impulsive, and combined, which are defined by the presentation and combination of symptoms (American Psychiatric Association, 2013). In most cases, the aetiology of ADHD is an accumulation of genetic and environmental risk factors (Faraone et al., 2021). ADHD is widely recognized as a childhood disorder that is most frequently diagnosed in boys, with estimates of the male to female sex ratio in childhood and adolescence (10–18-years old) ranging from 2:1 to 10:1, depending on a community or clinically based sample (Mowlem et al., 2019). Though it is commonly perceived as a childhood disorder, research efforts have explored the persistence of ADHD into adulthood (Waite, 2010). In adulthood (over 18 years of age), prevalence of ADHD diagnosis appears to be comparable between sexes, and, although symptom onset typically occurs in childhood, a significantly greater proportion of women receive a diagnosis in adulthood (Holthe & Langvik, 2017; Quinn & Madhoo, 2014). This has led to recent reports of a lack of ADHD diagnoses in females during childhood and adolescence (Quinn & Madhoo, 2014).

A potential explanation for underdiagnosed ADHD in females is differences in core symptomatic presentation between males and females. Research suggests that females are more disposed to internalized symptoms, such as inattentiveness and disorganization, and present with fewer symptoms of hyperactivity and other disruptive external behaviours (Quinn & Madhoo, 2014). On the other hand, males with ADHD are prone to more externalized hyperactive-impulsive symptoms, such as motor hyperactivity, rule-breaking and overt aggression, which results in greater likelihood of being identified and referred for clinical assessment (Abikoff et al., 2002; Young et al., 2020). As an internalized symptom profile is less likely to be disruptive in an educational setting, females are less likely to be identified and referred for clinical evaluation or treatment of ADHD. Furthermore, internalized symptoms, such as inattentiveness, are harder for the individual and others around them to identify. Symptoms such as “daydreaming,” emotional reactivity, or hyper-talkativeness may appear stereotypical of young females with the individual accepting these traits as their personality. These symptoms may also be interpreted by others as emotional or learning difficulties, instead of five symptoms of ADHD (Lynch & Davison, 2022).

Within the UK, clinical research surrounding ADHD lays focus to males, and, as a result, current diagnostic criteria and general understanding of ADHD is biased towards a male presentation of the disorder (Holthe & Langvik, 2017) with ADHD in females often being overlooked. Lynch and Davison (2022) identified that the Diagnostic and Statistical Manual of Mental Disorders Fifth Edition (DSM-V), used by clinicians to diagnose ADHD, contains a gendered bias that does not appreciate the subtle differences in the way females experience ADHD. The DSM-V lacks guidance on gender differences in ADHD, thus likely hindering the diagnosis of ADHD in females (Waite, 2010). In general, females with ADHD present with fewer of the symptoms outlined in the DSM-V compared to males, but are equally as impaired by their symptoms, though it may not be as visible (Quinn & Madhoo, 2014). In a consensus statement of ADHD in females, Young et al. (2020) emphasized the importance of moving away from preconceptions of ADHD as a disruptive behavioural disorder, and instead focusing on internalized presentations of the disorder, which are more common in girls and women.

The presence of co-morbidities in females also appears to cloud ADHD diagnosis. The overall prevalence of a comorbid psychiatric disorder for those with ADHD ranges from 40% to 80% (Shi et al., 2021). The most common psychiatric disorders that co-occur with ADHD include depression, anxiety disorders, substance use disorders, bipolar disorder, and personality disorders (Katzman et al., 2017). Specifically, females with ADHD are at a much greater risk of psychiatric comorbidities in comparison to males with ADHD and non-ADHD females (Solberg et al., 2018). Co-existing internalized symptoms of depression and/or anxiety have the potential to overshadow ADHD symptoms, resulting in missed diagnoses and therefore inappropriate treatment (Quinn, 2008). It is unclear whether psychiatric comorbidities, such as anxiety and depression, occur independent of ADHD or develop due to undiagnosed and untreated ADHD. Yet, these comorbidities have the potential to complicate identification and treatment of the disorder. Furthermore, in comparison to males, females with ADHD are more likely to develop effective masking strategies which hide the impact of their ADHD symptoms, resulting in problems being underestimated and their needs less likely to be met (Quinn & Madhoo, 2014).

Overall, ADHD is associated with significant likelihood of developing psychological, social, and emotional difficulties, which can be amplified when left undiagnosed and untreated. In general, ADHD is significantly associated with poor academic functioning across the lifespan, from school readiness to going to university (Daley & Birchwood, 2010). Problems at school are often the primary reason for a child’s clinical referral for diagnosis of ADHD, attributed to hyperactivity, inattentiveness, organizational difficulties, and lack of motivation (Arnold et al., 2020; Sedgwick, 2018). Poor educational outcomes can be a key predictor of long-term functional impairments, including anti-social behaviour, obesity, and social function outcomes, amongst others (M. Shaw et al., 2012). Among university students, poor academic attainment has shown to have a negative impact on students’ mental health and wellbeing (Sedgwick-Müller et al., 2022). There is evidence to suggest that females with ADHD do not present with academic difficulties until they reach higher education (Quinn, 2005). Quinn (2005) identified that girls with ADHD may work harder at school to mask their symptoms and meet parent/teacher expectations. However, in higher educational settings, such as university, it becomes increasingly difficult to cope with the struggles of ADHD amongst other lifestyle changes, such as moving away from home and taking on greater personal responsibilities (Quinn, 2005).

The potential absence of academic difficulties at school age may present greater challenges in recognizing ADHD in young females, providing further explanation for why many females do not receive a diagnosis of ADHD during their childhood. Individuals with ADHD commonly experience impaired social behaviours and interpersonal relationships (Sedgwick, 2018). University students with ADHD often face challenges making and maintaining academic and social relationships, and may struggle with participating in group work, team activities, and societies at university (Sedgwick-Müller et al., 2022). Impaired social functioning has a negative impact on self-esteem (Quinn & Madhoo, 2014). It is widely documented in the literature that girls and women with ADHD are more likely to experience low self-esteem in comparison to males with ADHD and non-ADHD females (Quinn & Madhoo, 2014). Rucklidge and Kaplan (1997) concluded that women who do not receive a diagnosis of ADHD until adulthood are at greater risk of suffering with low self-esteem. Low self-esteem predicts negative real-world consequences, such as unemployment, low socioeconomic status, and poor mental and 7 physical health (Trzesniewski et al., 2006). In a qualitative study exploring adult females’ experiences with ADHD, Holthe and Langvik (2017) identified that females with ADHD are more likely to experience psychological distress in comparison to males and non-ADHD females, due the profound social and personal impact of the disorder. Together, this highlights the severity of the impairments experienced by females with ADHD, exacerbated by a missed or late diagnosis of the disorder.

Overall, the literature highlights that there is insufficient understanding of ADHD in females, resulting in many females not being diagnosed with ADHD until they reach adulthood, and suffering with many of the consequences of a late diagnosis. Further research studies of females with ADHD are needed to improve understanding of the disorder and draw attention to the subtle differences in symptom presentation between males and females. Lynch and Davison (2022) highlighted that there is a lack of qualitative research exploring female experiences of ADHD from those directly affected by the condition. Sedgwick-Müller et al. (2022) also underlined that there is a deficit in research exploring the impact of ADHD on university students.

The aim of this qualitative research is to approach these gaps in the literature by exploring the personal experiences of adult, female university students with ADHD in the UK, and the impact of such experiences on their social, academic, and psychological functioning. This is an exploratory piece of research; therefore, the research does not address any specific research questions or hypotheses.

## Methodology

### Research Design

A qualitative approach was use, utilizing semi-structured interviews to gain an in-depth understanding of the personal experiences of adult female students with a clinical diagnosis of ADHD attending university in the UK. Semi-structured, in-depth interviews were identified as the optimal method for collecting qualitative data about participants’ experiences of ADHD (Creswell, 2007; Hammarberg et al., 2016). The General Health Questionnaire-28 (GHQ-28; Goldberg & Hillier, 1979), a self-report instrument widely used in assessing mental symptoms and psychological wellbeing, was implemented alongside the semi-structured interviews (Hjelle et al., 2019). The GHQ-28 was implemented to gain additional quantitative information about participant’s psychological wellbeing, and to explore potentially significant confounding variables, such as symptoms of depression and anxiety, which might have affected the experiences being explored.

### Participant Recruitment

A purposeful sampling method was used, recruiting participants based on a set of pre-determined criteria. The inclusion criteria comprised of being an adult female (18-years +), a student at any university within the UK with a clinical diagnosis of ADHD—no formal confirmation of participant’s diagnosis of ADHD was required. Exclusion criteria included participants who had a co-morbid diagnoses of another serious mental health disorder, such as a depressive disorder or a personality disorder. These exclusion criteria were selected as acknowledgement that diagnosis of another serious mental health disorder may present greater influence on the experiences being investigated in comparison to ADHD autonomously.

Participants were recruited via advertisements in several UK-based ADHD support groups for women on Facebook and Instagram. Twenty-five women registered their interest in taking part in the study, eight of which fully completed the consent forms, GHQ-9 questionnaire and attended their allocated interview time. Thirteen individuals did not provide full consent, three individuals did not return the questionnaire and one participant did not attend their interview. Eight interviews were conducted with intention of further interviews if data did not reach saturation. Following analysis of the data, it was agreed between the researchers that no further theoretical insights or themes would emerge from further interviews and that data had reached saturation (Charmaz, 2006).

The eight participants were adult women, aged between 22 and 53 years old (mean age of 31 years old). All participants were formally diagnosed with ADHD in adulthood, and either part-time or full-time students at a university in the UK. Further participant demographics, such as ethnicity or sexual orientation, were not collected, as this information was not relevant to the outcomes of the research.

### Procedure

Following obtainment of informed consent, participants were asked to complete the GHQ 28, which consisted of 28 short questions asking participants how their general health had been over the weeks prior to the study (Goldberg & Hillier, 1979). Participants responded to the questions based on four criteria: better than usual, same as usual, worse than usual, and much worse than usual. Goldberg and Hillier (1979) outlined four subscales of the GHQ-28 using factor analysis: somatic symptoms, anxiety/insomnia, social dysfunction, and severe depression. Each subscale contained seven questions. The validity and stability of this factor structure has been confirmed across several different contexts (Hjelle et al., 2019). Semi-structured interviews were conducted and recorded online via Microsoft Teams, a web-based video platform. All interviews were performed by AT. The interviews ranged in duration from 21 to 32 min, with a mean duration of 26 min.

Topics of discussion included: reasons for seeking a diagnosis, personal feelings after receiving a diagnosis, and the impact of ADHD on participants’ social, academic, and psychological functioning. Questions asked within the interviews were developed and agreed among the researchers to ensure the primary research question was approached and allow an easier triangulation process. A full outline of the interview questions used can be found in Appendix A. Participants were encouraged to elaborate on topics which they found relevant. Following completion of the study, participants received a £5 Amazon e-voucher as reasonable compensation for their time.

### Data Analysis

Participants were scored on their responses to the GHQ-28 using a Likert scale scoring system of 0 to 3 (0 = better than usual, 1 = same as usual, 2 = worse than usual, 3 = much worse than usual). Mean scores and standard deviations were calculated for each of the four subscales of the GHQ-28. The lowest possible total subscale score is 0 and the highest subscale score is 21. Lower scores indicated lower levels of psychological distress, and higher scores indicated higher levels of psychological distress amongst participants. All interviews were transcribed and analysed by one author and checked by the other. A full transcript of the interviews can be found in Appendix B. Content of the interviews was analysed in accordance with thematic analysis, chosen as an accessible and flexible method for identifying and interpreting themes and patterns of meaning within qualitative data (Braun & Clarke, 2006; Clarke & Braun, 2017). The six steps of thematic analysis include familiarizing yourself with the data, generating initial codes, searching for themes, reviewing themes, defining, and naming themes, and producing the report (Braun & Clarke, 2006). An inductive approach was used to analyse the data and generate codes and themes.

### Data Triangulation

Both authors independently analysed the raw data and developed initial codes and themes. These codes and themes were then compared between the researchers referring to the raw data to ensure validity. Any discrepancies of themes and codes developed were discussed with agreement that a third researcher would be involved if an agreement could not be made. There were no discrepancies between the codes and themes made.

### Ethical Considerations

Ethical Approval was obtained from the University of Nottingham Research Ethics Committee (Ref: S1439R). The ethical considerations reviewed for this study centred around confidentiality, and implications of the discussion of personal memories surrounding a problem which had received clinical psychological attention. Informed 10 consent was required from all research participants. Participants were informed that the researcher was not a clinical professional, therefore could not provide any form of diagnosis or psychological care. At the end of the online interviews, participants were fully debriefed and provided links to professional support services, both within the University of Nottingham and outside the University of Nottingham, should participants have felt affected by the nature of the interview and/or discussion of sensitive topics. To maintain confidentiality, all identifiable information was removed from the data following transcription and names were replaced with pseudonyms.

## Results

Mean scores and standard deviations (SD) for each of the subscales of the General Health Questionnaire-28 (GHQ-28) are outlined in Table 1. Participants scored higher in domains of anxiety/insomnia, and social dysfunction, compared to the other subscales, arguably due to the nature of ADHD and comorbid conditions. However, overall, the scores do not indicate high levels of psychological distress amongst participants. Therefore, it is unlikely that the subscales of the GHQ-28 would have acted as significant confounding factors in the experiences being investigated. Thematic analysis of the raw data identified three core themes and six sub-themes. Derived from the coding framework, the themes and sub-themes provide insight into participants’ experiences of ADHD as a female university student in the UK. The three core themes, which guide the order of presentation of the findings, include “stigmatization 11 surrounding ADHD in females,” “journey to receiving an ADHD diagnosis” and “adulthood diagnosis.” The structure of the core themes and defining subthemes are described in Table 2.

**Table 1.** Mean Scores and Standard Deviations of the Subscales of the General Health Questionnaire 28.

	Somatic symptoms	Anxiety/Insomnia	Social dysfunction	Severe depression
Mean	6.88	9.88	9.38	1.75
SD	3.31	4.05	2.07	2.7

**Table 2.** Structure of Main Themes and Defining Subthemes.

Main Themes and Defining Subthemes	
Stigmatization surrounding ADHD in females	Misconceptions, social discrimination, and prejudice Selective disclosure
Journey to receiving an ADHD diagnosis	Symptoms affecting academic, social, and psychological functioning Prompted to seek a diagnosis by others
Adulthood diagnosis	Mixed emotions Emotional validation

## Theme 1—Stigmatization Surrounding ADHD in Females

### Misconceptions, Social Discrimination, and Prejudice

Female adults suffering with ADHD are at a high risk of being confronted with stigmatization due to the lack of disorder-related understanding and male stereotypes surrounding ADHD, amongst other factors (Mueller et al., 2012). Topics of stigma, public misconception, prejudice, and discrimination recurred throughout participants’ experiences. Most participants commented on the general lack of understanding and public awareness of ADHD, particularly surrounding the differences between ADHD in males and females. Participants acknowledged that ADHD is often perceived as a disorder that affects hyperactive teenage boys. One participant experienced prejudice during their assessment for ADHD as their symptoms did not align with the male stereotype of the disorder.

*“I felt like I wasn’t taken seriously during my assessment. . .because I arrived really early, which the person assessing me kept bringing up, like, well you arrived on time so obviously that’s not a problem for you. And they were making comments like I wasn’t fidgeting enough, at least in their eyes. And those kind of comments knocked my confidence and I felt like I wasn’t being believed”*

Similarly, one participant was told that she was depressed during her initial ADHD assessment as she was overwhelmed and visibly emotional. The participant knew that she was not depressed, and so felt that her feelings and experiences were being dismissed by the GP. Young et al. (2020) highlighted that it is not uncommon for adults with ADHD to be treated for depression in the first instance, as affective symptoms, such as emotional dysregulation, are often misattributed to depressive disorders. One participant reported experiencing discrimination at work, which had a serious impact on her mental health and resulted in leaving her job.

*“People [at work] were making comments and I was getting into trouble for things that I now realise were symptoms [of ADHD] . . .and I felt like it was really unfair, and it was messing with my head a little bit”*

Furthermore, several participants also described experiencing negative attitudes towards ADHD, from family members and academic staff. Participants expressed shock over the stigma surrounding ADHD that they faced.

*“I had one tutor at university who didn’t believe in ADHD and dyslexia and that, she just believed it was a lazy excuse for not going to school”*

*“When I told my auntie [about my diagnosis], she was really unsupportive, and I was really shocked. She says she knows a lot of kids with ADHD and autism and says it is brought on by their diet and that makes them go crazy when their parents give them processed food”*

One participant explained that they did not wish that they had their diagnosis sooner, as they felt that they would have faced even greater public misconception. Overall, participants remarked that the lack of understanding and awareness of ADHD in females is what led to their ADHD being undiagnosed throughout their childhood. However, some participants did acknowledge that awareness of ADHD in females is improving, but there is still a long way to go.

### Selective Disclosure

As a result of the stigmatization and negative misconceptions surrounding ADHD, some participants avoided telling close friends, family, and colleagues about their diagnosis. Participants described that they felt like they would be judged and perceived differently for having ADHD.

“Even now, I don’t tell many people in my personal life about my diagnosis. I am a bit more open about it at university, but I am worried about what some family members and close friends will think of me if I tell them about it” “Now, like I don’t really tell people [about my diagnosis], like I tell my close friends and family, but for work, it’s very much a need to know basis, because I think, if you’re female, most people will just go right well you don’t have ADHD and you can’t be bothered explaining yourself really, so there’s just no point”

## Theme 2—Journey to Receiving an ADHD Diagnosis

### Symptoms Affecting Academic, Social, and Psychological Functioning

Prior to receiving a diagnosis, participants described struggling with various aspects of daily life, without realizing that their struggles were consistent with symptoms of ADHD. For many participants, these problems were most prevalent in an academic setting, primarily due to the inability to focus and maintain concentration. It has been widely documented in the literature that inattention, a core symptom of ADHD, is associated with poor academic functioning (Sedgwick, 2018). Several participants remarked that, although they did not do badly at school, they struggled with motivation, particularly in subjects that they did not enjoy.

*“I was great in the subjects that I was good at, which was usually the more creative ones like, you know, the more practical subjects like PE, drama, and art, and I did really well in those. And it’s not to say I did really badly in other subjects, I was capable of doing it, but I just couldn’t focus to do it, so I refused to do the work”*

Furthermore, most participants described having problems with time management and procrastination, and struggling to organize their thoughts, which acted as a barrier to performing well academically. Before receiving a diagnosis, many participants described that they felt that these symptoms were part of their personality and felt that they were “simply just not academic.” Though, not all participants struggled with academic performance; one participant described being a perfectionist and seeking a lot of self-worth and validation from her academic work, which led to overworking herself at school from a young age.

*“If I didn’t do well on a test at school, I would completely have a meltdown, for me it was like the end of the world. . .I remember being at a parents evening when I was 11 years old, and the teacher told my parents that I was on the verge of burnout if I kept working as much as I was, which, you know, should not be happening to an 11 year old”*

Consistent with the literature on ADHD, a recurring theme throughout all women’s experiences was suffering with low self-esteem, which had a profound impact on participants’ social, academic, and psychological functioning (Trzesniewski et al., 2006). Many participants described comparing themselves to other people around them, which left them feeling like “*there is something wrong with me*.”

*“I really felt myself being inferior to other people, I suppose, and I always had a feeling that I wasn’t good enough. You know, you’re thinking what is wrong with me? Why can’t I do that? Why can’t I be as organised as other people? How do they do it?”*

One participant described believing that “*people had to put up with me, instead of enjoying my company, and I felt like I had to apologise for the way that I was*,” which had a huge impact on their self-perception. Fifteen Linked to low self-esteem, many participants also described issues specifically related to their social functioning, including problems creating and maintaining friendships. For example, one participant described having “*lots of acquaintances, but not very many close friends*” as they struggled to hold onto close friendship groups, and often found that they would neglect friendships and relationships when they were busy or stressed. Some participants also described having problems with emotional dysregulation, which had a large impact on their social functioning.

“*One of the biggest things that affected me was my emotional sensitivity, and like fear of rejection, like if a friend didn’t text me back, I thought they hated me. . .I think I would have benefited when I was younger knowing about that emotional side, as that really had a huge impact on my friendships and relationships which was pretty difficult*”

Emotional dysregulation is a very common symptom of ADHD, often more severe amongst females (P. Shaw et al., 2014; Young et al., 2020). An aspect of emotional dysregulation, called rejection sensitive dysphoria (RSD), was described by several participants. RSD causes individuals to feel intense emotional pain from feeling as if you have failed to meet your own or other’s expectations (Bedrossian, 2021). RSD, though not widely recognized, is estimated to affect 99% of adults with ADHD (Bedrossian, 2021). Despite the social issues that participants faced, most participants described having a strong support network of family and friends with whom they could share their experiences and seek emotional help.

### Prompted to Seek a Diagnosis by Others

Many participants described that they were prompted to seek a diagnosis by a member of their support network, such as a friend, family member, or colleague, who had recognized that they were presenting with some of the symptoms of ADHD. Despite acknowledging that they were struggling with certain aspects of their life, most participants commented that they did not realize that this could have been due to ADHD, as they were not aware of many of the symptoms of ADHD, particularly in females. A couple of participants reported that they had their suspicions that they might have ADHD but chose not to act on it. For 16 several participants, ADHD had been mentioned in their childhood, but, at the time, it was not taken seriously enough to seek professional help.

*“I’ve got a friend who has ADHD, and she noticed a lot of similarities between us, and said it seemed like something I should follow up with. I sort of had a suspicion [that it might be ADHD], but I didn’t bother following up with it because it seemed like a lot of work, and at the time, I didn’t think it was impacting my life that much”*

Some participants also disclosed that they had a comorbid condition, including dyslexia, autism spectrum disorder, or anxiety, and a history of issues with self-harm and eating disorders. Comorbidities were highlighted by several participants as a possible reason why they never realized that they also had ADHD. The presence of comorbidities in this sample are consistent with the literature, highlighting that comorbid disorders present possible overlapping symptoms with ADHD which can create challenges for diagnosis (Katzman et al., 2017). Seven out of eight participants referred themselves for a diagnosis after they researched more about ADHD and realized that they would benefit from seeking professional help.

## Theme 3—Adulthood diagnosis

All participants received a diagnosis of ADHD in adulthood. For some participants, the diagnosis process was relatively quick and easy and lasted a matter of 3 months, however, for others, it took up to 2 years.

### Mixed Emotions

After receiving a diagnosis, participants reported very mixed emotions. Most participants described feelings of relief and recognition. One participant described feeling like “*a weight had been lifted off their shoulders*,” as they felt that there was an explanation for why they had been struggling in many areas of their life.

*“For me, there was a lot of relief. I mean, I had spent my whole adult life thinking that I just wasn’t good enough, and I kept falling short in a lot of areas of my life, I 17 thought I was lazy, I thought I was useless. . .so hearing that it wasn’t just me and there is something else going on, I guess, yeah, it was a huge relief”*

However, not all participants felt a sense of relief following diagnosis. Despite acknowledging that diagnosis was a positive thing which meant that they would be able to get support, one participant commented that they “*didn’t feel anything*.”

### Emotional Validation

Although there were mixed emotions initially, all participants described feelings of validation once they had time to think about their diagnosis. Participants were able to understand themselves better and noted that looking back, their behaviour “*made sense*.” Validated feelings and experiences improved participants’ self-esteem, and many participants reported being kinder and more compassionate to themselves following diagnosis. Furthermore, many participants implemented appropriate management strategies, such as seeking professional support and using good organization. For many participants, stimulant medication had been the most useful management strategy. Likewise, having a better understanding of their strengths and weaknesses meant that participants felt they had better control over their lives.

*“Having a diagnosis has changed the way I approach lots of things, I plan things differently, I’m aware that I might struggle with certain things, and I don’t beat myself up if I can’t do things”*

Since diagnosis, many participants returned to full-time education and started university, as they were able to approach things differently this time round. Participants generally reported receiving lots of support from their university, which made the experience of returning to education much easier for them. Some participants reported feeling like they needed to prove to themselves, and others, that they can do it. Participants described a need to fulfil their potential by going to university, and pursuing the career that they always wanted, but felt they were unable to achieve prior to being diagnosed with ADHD.

*“I’ve spent my whole life thinking that I wasn’t capable [of going to university], and now I’ve finally got the belief and motivation behind me to do it. It’s almost like I’m 18 trying to prove something just to myself and everybody else that’s doubted my ability”*

All participants benefited from receiving a diagnosis. However, there are still daily challenges that they face with ADHD. Participants explained that they have good and bad days, and with each day, they are constantly learning new things about themselves.

## Discussion

The personal experiences of female students with ADHD explored in this qualitative study are rich and insightful, highlighting the profound impact of ADHD on social, academic, and psychological functioning.

The themes and sub-themes identified in this study overlap with previous literature surrounding females with ADHD. Many of the symptoms which affected participants academic functioning correspond with the literature review of ADHD in university students presented by Sedgwick (2018), including inability to focus, easily distracted, boredom, and lack of motivation. However, despite struggling with these symptoms and despite the wealth of literature which suggests that ADHD is associated with poor educational outcomes (Arnold et al., 2020; Daley & Birchwood, 2010; Sedgwick-Müller et al., 2022), most participants stated that they did not perform badly when they were at school (ages 5–16). As mentioned previously, one participant described that she was a perfectionist from a young age, driven by the validation of receiving good grades. This is in line with the research presented by Quinn (2005), highlighting that not all females with ADHD struggle with academic performance at school, as they often work harder to compensate for their symptoms and meet parent/teacher expectations. Greater challenges may present when an individual goes to university, as it becomes harder to cope with the struggles of ADHD amongst other lifestyle changes, such as moving away from home and having greater personal responsibilities (Quinn, 2005; Sedgwick-Müller et al., 2022). At the time of interviewing, all participants were at least 1 year into an undergraduate university degree, some even studying at master’s or doctorate level. With appropriate recognition, support, and management for their ADHD, participants were able to perform well academically and achieve their long-term goals by going to university. However, participants explained that they would often receive comments such as “you have a degree; you can’t have ADHD,” amongst other false claims that individuals with ADHD do not perform well academically. This highlights that, despite the literature and common perceptions, which often overlooks females, ADHD is not solely characterized by poor academic performance and poor educational outcomes.

Intense feelings of low self-esteem resonated throughout participants’ experiences. Poor self-esteem is very common amongst females with ADHD (Holthe & Langvik, 2017; Quinn, 2005), triggered by feelings of failure and “not being good enough.” Feelings of 20 inadequacy are exacerbated by other’s perceptions and expectations and ultimately, by an absence of an ADHD diagnosis throughout childhood. Low self-esteem has a knock-on effect on an individual’s social, academic, and psychological functioning. Participants explained that having low self-esteem negatively impacted many different aspects of their life, including body image, eating habits, confidence at school, romantic relationships, and close friendships. As outlined in the sub-theme of “emotional validation,” participants’ self-esteem improved significantly following diagnosis, as participants reported understanding themselves and their needs better, which allowed them to feel much more in control of their lives. It is evident that receiving a later diagnosis in adulthood had a profound negative impact on participant’s wellbeing throughout their childhood. This emphasizes the importance of earlier recognition and diagnosis of ADHD in females.

In accordance with the literature, participants also faced problems forming and maintaining friendships, particularly at school (Sedgwick, 2018). Despite this, most participants described having a strong support group formed of close friends and family members whom they could rely on for social and emotional help. A strong support group was further evidenced by the subtheme of “prompted to seek a diagnosis by others,” in which, for most participants, the possibility of having ADHD was first raised by someone within their support group. This further highlights the importance of disorder-related understanding, as with greater and more widespread understanding of ADHD in females comes greater recognition and diagnosis for those who need it.

The aim of the research was to explore the impact of ADHD on participants’ social, academic, and psychological functioning, as discussed in the themes of “journey to receiving an ADHD diagnosis” and “adulthood diagnosis.” However, following data analysis, the theme of “stigmatization surrounding ADHD in females” emerged from the data and became a highly influential element in participants’ experiences of ADHD. Most participants described at least one personal experience of public misconception, social discrimination, or prejudice. Stigmatizing attitudes towards both children and adults with ADHD are very common (Mueller et al., 2012). As a disorder, ADHD is vulnerable to stigmatization due to its largely unknown aetiology; there is not a single causal factor, nor a single diagnostic test used to identify ADHD. As a result, there are many public misconceptions about the origins of the disorder, including myths that ADHD occurs in children who eat too much sugar, or that it is caused by poor parenting (Mueller et al., 2012). These public misconceptions and stigmatizations are strengthened by a lack of disorder-related knowledge.

There is even greater stigmatization towards females with ADHD, in comparison to males, as it is publicly recognized as a disorder that predominantly affects boys. Schmitz et al. (2003) observed that the stereotypical profile of an individual with ADHD is described as “a young, white, middle-class boy suffering with hyperactivity.” Due to this common misperception, participants were often told that “they can’t have ADHD” or that “they aren’t hyperactive enough.” Furthermore, as confirmed by several participants in this study, females with ADHD often experience prejudice and social discrimination. For females, their disorder is often not taken seriously enough and they “feel like they aren’t being believed” by those whom they share their diagnosis with. McKeague et al. (2015) investigated experiences of stigma in young people with ADHD and observed that stigmatization from others results in negativity towards the self, called “self-stigma.” Self-stigma is an individual’s recognition that others hold prejudice and will discriminate towards them due to their disorder, which can severely affect one’s self-esteem and psychological wellbeing (Corrigan & Rao, 2012). Consequently, many participants reported that they are selective with whom they share details of their diagnosis with, in fear of being judged or perceived differently, or because they “don’t want to have to explain themselves.” This finding is in line with the findings of Holthe and Langvik (2017), who also identified the theme of “to tell or not to tell: selective disclosure” in their thematic analysis of adult women’s experiences with ADHD. This demonstrates that it is common amongst females with ADHD to withhold their diagnosis of ADHD from others, primarily due to the stigmatization and public misconceptions surrounding ADHD in females.

Overall, the women’s experiences discussed in this qualitative study draw attention to the complexity of ADHD. The heterogeneity of symptom presentation presents many challenges in recognizing and diagnosing ADHD in females. However, the overarching theme amongst all participant’s experiences is a clear lack of understanding of ADHD in females. Even amongst health professionals, there is a lack of appreciation of the differences in symptom presentation between males and females with ADHD, and an overwhelming existence of stigmatization. This lack of disorder-related knowledge, 22 alongside the presence of comorbidities, plays a key role in females receiving a late diagnosis of ADHD.

### Limitations

This research responds to gaps in the literature by exploring several rich and detailed accounts of first-hand experiences of ADHD in adult female university students in the UK. However, there are number of caveats with the sample used in this study. Firstly, given that females with ADHD are largely underdiagnosed in the general population, the sample of formally diagnosed females used in this study may not be fully representative of females with ADHD in the wider population (Lynch & Davison, 2022). It is likely that many females in the general population have ADHD that is undiagnosed, possibly due to having less severe symptoms, or have reduced access to support, in comparison to the females in this sample. Additionally, as the participants used in this study were diagnosed during adulthood, their experiences will likely differ from those who received a diagnosis in childhood (Lynch & Davison, 2022).

Another caveat of the sample used in this study is the exclusion of participants who have comorbid diagnoses of another serious mental health disorder, such as a depressive disorder or personality disorder. This presents a severe limitation to the research as there is a high incidence of depression, amongst other psychiatric disorders, in females with ADHD (Solberg et al., 2018). Therefore, the results of this study are not representative of the entire population of females with ADHD. Possibly linked to the exclusion of participants with a serious mental health disorder, the results of the GHQ-28 identified that the participants in this sample were all in relatively good physical and psychological health. Thus, participants’ experiences will likely differ from those who are suffering with their health. Overall, the results of this study do not provide a complete picture of females with ADHD and should not be generalized to all contexts.

### Implications and Directions for Future Research

Despite the addressed limitations, this research provides meaningful insight into the experiences of women with ADHD. Today, many women with ADHD experience stigmatization and social discrimination. Therefore, the clear take-home message of this study is the need for greater public understanding and appreciation of ADHD in females. Twenty-three Improving public knowledge of ADHD is important moving forwards to reduce stigmatization and debunk stereotypes of ADHD as a disorder that predominantly affects males.

Furthermore, greater understanding of ADHD is particularly important in allowing for earlier diagnosis of the disorder. Earlier diagnosis allows individuals with ADHD to develop effective management strategies, which in turn improves their social, academic, and psychological functioning. Greater public understanding of ADHD in females can be achieved through education. Moving forwards, particularly amongst health professionals and teachers, ADHD should be treated as a disorder that affects both males and females, and attention should be drawn to potential differences in symptomatic presentation between males and females.

However, ADHD in females should not be treated as separate to ADHD in males, as this can feed into further stereotypes. The findings of this study identify the need for larger scale research surrounding ADHD in females, to strengthen the validity of this research. In conclusion, the results of this qualitative study provide significant insight into the complex and challenging nature of ADHD amongst female university students in the UK.

## Appendix A

### Outline of Interview Questions

Firstly, could you tell me about yourself and your journey to receiving an ADHD diagnosis? (Prompts: when did you receive a diagnosis and what led to you seeking a diagnosis of ADHD?)

How did you feel after receiving a diagnosis?

How has having ADHD impacted experiences related to your social functioning? (Prompts: such as your relationships/friendships/social activities).

How has having ADHD impacted your academic functioning?

(Prompts: such as your performance at school/university, organization, meeting deadlines, did you feel supported at school/university?).

How has having ADHD impacted your psychological functioning? (Prompts: such as general wellbeing, emotional control, self-esteem etc.).

Finally, is there anything else you wish to discuss which you feel has been relevant to your journey of ADHD?

## Appendix B

### Full Transcript of Participant Interviews

#### Interview Participant #1

Interviewed by Aimee Tyrrell on 10/08/2022. Interviewee pseudonym: Sophie.

##### A: OK. So, to start with, could you tell me about your journey to receive your ADHD diagnosis. For example, when did you receive a diagnosis and what led to you seeking a diagnosis?

S: So, it wasn’t until I went to uni that I realized that I had a lot of ADHD symptoms. I didn’t go to uni until 2020, I didn’t go straight after sixth form. I started to realize that I had a lot of the symptoms, especially with like online learning and when we hit the pandemic. But there was such a waiting list for the NHS to get diagnosed, so I was on the waiting list for a while. I was on it for over a year and I kind of wasn’t getting anywhere, so I went privately and only got diagnosed at the end of June this year, so literally just like really recently. But I kind of knew I had it for ages, but obviously like I didn’t have an official diagnosis. I got diagnosed in June of this year and I have just started medication as well. So, I have been on medication for about 6 weeks now, so still very early.

##### A: How did you feel after receiving that diagnosis?

S: Umm, it definitely was a relief, definitely. I mean, it’s a really hard thing at the moment because obviously the NHS waiting list is so long, but equally privately was like horrifically expensive. So, it kind of does leave you in like a really hard situation. But once I had got the diagnosis, I was so relieved that I’d finally like, kind of been recognized, and you know me and my psychiatrist who diagnosed me, you know, spoke about my childhood and, like, how I’ve been at school and it kind of all made sense. And I finally realized why, like, this is why I’ve been struggling with XY and Z, like at school, because I had it all along. My psychiatrist reckons I had it from between the ages of 6 and 12, and I’m obviously 22 now. So, that’s a long time to go without realizing I had it. So, it was kind of more relief than anything.

##### A: How has having ADHD impacted any experiences related to your social functioning? Do you think it was affected your relationships and friendships?

S: Umm, I think now that I have been diagnosed, I realize the way I have been socially makes sense. So, for example, like I’ve always tried to be a good friend, but I am one of those people that is like awful at replying on text and stuff, and now I realize that it’s just because, you know, like the way my ADHD brain works and like, very all over the place. And so, it doesn’t make me a bad friend, it’s just that’s the way I am. So, I think it kind of just makes sense now. Like, the way I am with a friend depends on how I’m feeling with myself. So, if I have got a lot going on and for me that like really stresses me out, then I will just kind of like disappear off social media and like not reply to messages and stuff. Whereas if I feel like good in myself and I feel like I’ve got everything under control, I will be all into my friendships, if that makes sense.

##### A: How has having ADHD impacted your academic functioning?

S: Yeah, I think the academic side of things is the main thing that made me realize I had it. One of the main things I suffer with and one of the main things that girls really suffer with is task avoidance. Like, if anything gets too stressful, I end up completely putting it off until like, I actually cannot put it off anymore. And that has always been me. And at first, I thought, ohh, you know, everyone procrastinates, things like that. But mine was really severe until the point where I would actually make more work for myself because I would like push back a deadline and ask for an extension. But then I would have so many extensions that I would actually be making more work for myself. And also, a big thing with ADHD is like making a mountain out of a very small task, so, say I had like an essay to do, or, you know, like when you’re at school and you have to make a poster about something. Something that could have taken an hour would take me like 5 hr because I would just like overcomplicate it and make it so much more than it actually had to be. Like, I would always leave something until the 11th hour, even though I’ve had two weeks to do something, I’ll always be that person that is still doing something right up until the deadline. Sometimes it is the other way round, as I avoided a task for so long, I could then do like 6 hr of work in 2 hr if I had to. And that is actually like a form, now that I actually have a diagnosis of ADHD I can understand it a bit more, and that is like a form of masking, and there is a lot of ties between ADHD and perfectionism, and that was absolutely me. Even if the work is done, I will still be working until the deadline because I’m like I have to use all the time that I have before I submit it, otherwise in my head I haven’t done enough.

At kind of school age, if I like didn’t do well on a test that didn’t even mean anything, I would completely have a meltdown, and I kind of used to think like, you know, c’mon [Sophie], like, put it into perspective, this is one test, it’s not going to determine everything. But for me it was just like the end of the world because I was like, such a perfectionist. I didn’t struggle in terms of being able to do the work, but it was just like the deadlines and things like that that sent me, like, into meltdown. It is a really hard situation to be in, like being a perfectionist, but then also putting stuff off until like I have to do it.

##### A: How have you found managing your academic work since being diagnosed and understanding your ADHD?

S: As soon as I went on medication, I just started to organize myself more. I had energy to get things done and I think like the first week I was on medication I got things done that I’d literally been putting off for like months and then I got them done in like half an hour and I was like, oh, I could have done that six months ago. But like I said, I’ve got this extreme task avoidance where I would put something off. And I would like justify in my head why I put it off as well. I’m like ohh that doesn’t need to be done today, I’ll do it tomorrow, but then tomorrow comes and I just won’t. But since being on medication I just get things done. So, it’s almost like something kind of like turned as soon as I had medication, and like I know now it is because of the dopamine hit you get when you go on medication, it gives you that kind of spur of energy to do things, and I’ve definitely found that.

##### A: Did you feel like there was a lack of support at school/university?

S: I definitely, like I said, was struggling throughout school. I was like a huge over worker from such a young age. I remember like, being like 11 years of age and my teacher saying to my parents at parents evening, like she will burn herself out if she keeps working this much. And if you think about it, like an 11-year-old should not be burnt out. In this case, this was not a particularly important stage of school in terms of like A Levels and GCSE, but I was overworking, and I was that quiet child that didn’t really say much in class but was like completely having a meltdown about everything. Umm, but I mean, I don’t necessarily like blame schools for not picking it up, because I generally think that, up until recently, nobody really knew what ADHD was or how it presented itself, especially in females. I think the stigma about ADHD, just being that like annoying boy at school that wants attention, still very much exists and so I think when I was 11 and I was told that I was on the verge of burnout, I don’t think anyone would at any point have thought, oh, she’s got ADHD. Like that’s her way of masking by overworking. I honestly just think that they thought I was that student that just worked really hard, but I don’t necessarily blame schools. I just think that there was at the time no kind of awareness of what ADHD really was. But I think maybe moving forward, there definitely needs to be more discussion about it in schools and stuff, because so many girls that I’ve kind of connected with on social media that have ADHD are very much the same. Like nobody picked up on it. Nobody knew about it.

##### A: How has having ADHD impacted your psychological functioning, for example, your self- esteem?

S: Yeah, I think, again, academically, and being that perfectionist and overworking and then striving for academic validation. And if I didn’t get that, I would completely like, lose confidence, self-esteem, because I’ve kind of put all of my self-worth into academic stuff. And now that I have been diagnosed with ADHD, I realize that this is an ADHD trait. And I’m obviously not at that point now where I think that my worth is like completely academic, that was very much when I was like maybe 16 at GCSE age. But definitely back then, definitely.

##### A: Is there anything else that you want to talk about that is relevant to your experiences of ADHD?

S: Not that I can think of, but I think it’s really great that you’re doing this because like I said, there is like not a lot of research about ADHD and awareness about girls with ADHD, so yeah, it’s really good what you’re doing.

#### Interview Participant #2

Interviewed by Aimee Tyrrell on 15/08/2022. Interviewee pseudonym: Lucy.

##### A: Could you please tell me a bit about yourself and your journey to receiving an ADHD diagnosis?

L: So, I guess my whole kind of life I’ve kind of struggled with a lot of things, including focus and motivation, and it had kinda been spoken about over the years, jokingly, that I might have ADHD. And I went through a really rough patch in my life, which meant that my mental health declined, and so when I was coming out of that, I did a lot of self-reflection and trying to improve myself. And it was during that time of self-reflection that I realized that I was still struggling and that there was areas that didn’t improve no matter how hard I worked on them. I still kept falling short and not meeting the mark. And that is what kind of pushed me more into getting an ADHD diagnosis, and then along with that, a friend of mine, had a friend who had also just been diagnosed and when she was explaining herself, she said that essentially it sounded like she was explaining my life, it sounded like she was talking about me. So, my friend brought that up and she’s like have you ever thought maybe about doing more research into a diagnosis, and that’s what kind of prompted me to go and to look further into it, which is what I did. Yeah, so I’m still waiting for an official diagnosis from the NHS, but I have a diagnosis of such through the university I went to. I approached the inclusions team when I started uni and just told them what I was going through and they told me they could put me to an educational psychologist and essentially assess me for ADHD themselves, which is what I did. So, I kind of, that was in September last year, just towards the end of first year, so, so yeah, I started uni last year, I decided like it was around the same time that I was actually looking for the ADHD diagnosis, and I thought, you know, if I have ADHD, it will explain a lot of why I have never had the courage to go to university before. I just put it down to the fact that I wasn’t academic, I just had that, that was what I convinced myself. I just thought that I wasn’t academic, and now knowing that there is something else kind of going on that was like, well, right, if I have ADHD, then maybe I can do university, maybe I can do this, I just need to think about things a little bit different and get the support I need. So, that is what kind of pushed me to apply.

##### A: How do you feel knowing you have ADHD?

L: I think, for a start, there was a lot of relief. Because I mean, I spent my whole adult life thinking that I just wasn’t good enough and I just wasn’t, you know, like, because I kept falling short in a lot of areas in my life, I thought I was lazy. I thought I was useless. I just, I spent my whole life thinking that, you know, why are all these other people managing to do, to do these things? And I can’t do them, and I just put it down to myself being really poor at life, and so hearing that it wasn’t just me and that there is something else going on, was like I guess, yeah, it was just a huge relief. It was just almost this weight had been lifted off my shoulders, just to know that it’s not actually your fault, there is something else at play here. So yeah, massive, massive relief. It was hugely validating for me to find out from the university that it was ADHD, and just, yeah, it’s taken a lot of years of self-loathing kind of away. I mean it’s not, you can’t just overcome that, you know, all of a sudden, but it’s definitely helped, and I am definitely a lot kinder to myself now because of that.

##### A: How would you say ADHD has impacted your social functioning?

L: Yeah, I mean, I think because of the self-esteem issues, it has affected me socially because I’ve become a people pleaser. And so, a lot of the time, rather than just being myself and sticking to my values, a lot of the time I’m trying to meet the values of others around me. And so, I find myself people pleasing a lot to make other people happy, sometimes at the expense of my own happiness. But again, like since diagnosis that’s probably improved a little bit more and I’m definitely kinda, looking after myself a little bit more and sticking to my values in life a little bit better. I’d say, before my diagnosis, I just thought it was the way I was, I mean, my parents split up when I was young as well, so I thought there was potentially like, you know, a little bit of trauma there from my childhood and that had a part to play in it, which it will be a part to play in all of that, but yeah, I just thought it’s who I was. I’m just like, maybe I’m just not able to excel in anything in particular, and you know, I’ve always seen myself as a person, that’s like, good at lots of things, but not really great at anything. And I guess, through learning about ADHD, it’s just like, well that’s like I am good at lots of things, but the reason that I never really get great at anything is because once it gets difficult or I need to focus more, is when I fall kind of by the wayside.

##### A: How would you say ADHD has impacted your academic functioning?

L: Well, the inability to concentrate has had a massive effect on my life, because I struggled, I think I probably became a bit rebellious as well, and then I was a little menace when I was at school. I was great in the subjects that I was good at, which usually was the creative ones and like you know, the more, what’s the word, the more practical things, like home economics, drama, PE, art, all of those things I did really well in. And it’s not to say that I didn’t do well in other subjects, when went and I did my standard grades, I was in the top class for maths. But because of the work that I had to put into it, I just wasn’t able to do it because I just, now looking back, it was the inability to focus. And so, I just refused to do the work because I was like, I can’t do it, which I could. I was capable of doing it, but I just couldn’t focus to do it, so I essentially like, pushed myself down to make things easier for myself. So, I went from being the top class at maths, and I ended up coming down into the second class and then trying to get down into the third class just so I could make life a little bit easier for me. And I think that was definitely affected by my undiagnosed ADHD because I know that I was capable and I guess a lot of my teachers said that and in all my reports, they said I was great when I was doing practical work, even in the likes of biology and stuff, I was really good when I was engaged and doing practical stuff, but I really struggled when we had to sit down and concentrate and write and or do homework. I just wasn’t able to do it. I wasn’t able to self-direct because I just didn’t have the ability to focus to do things on my own. So, I really struggled when it came to that stuff just because of the lack of focus.

##### A: How is it at university now?

L: When I started uni in the first semester, I was really focused and I did really well and I finished first semester with A’s and I was just like, you know, super, super happy and really proud of what I was doing. And umm, but I see that in a lot of things that I do, it’s like I’ve been to college and stuff before in the past and I always go in with so much enthusiasm and, don’t get me wrong like the want to do well is still there just as much as it was from the start, but it’s the ability to keep that momentum and to keep that focus and motivation going that I struggle with. So, when I went back for second semester, I could see myself kind of declining slightly and that starts to frustrate me because I know I can do it, but I just can’t keep the momentum and the focus up. So, I didn’t do as well second semester, I mean I got a couple of A’s and I think there was a B and a C in there as well, but I knew I could have got those A’s, but I was just struggling with my focus and motivation. So, and this is where it’s frustrating where I want my diagnosis and the help in place so that I can obviously get through uni cause I want to go back in second year and I want to do well and like I’ve spent my whole life thinking that I wasn’t capable of doing this and now I’ve finally got the belief behind me to do this and that I can do it and, it’s almost like I’m trying to prove something just to myself and everybody else that’s doubted me and my ability. So yeah, so like it is still a struggle, but the uni has been really helpful in that. Obviously, I got my diagnosis through the psychologist there, and on top of that, they’ve put things in place to help me and, and I’ll get support throughout now that they know I have ADHD, they are gonna offer me support throughout which, which feels good and makes me think, you know, they don’t want me to fail either. So, they’re gonna help to make sure that doesn’t happen, which is really good.

##### A: How has ADHD impacted your psychological functioning?

L: Yeah, well, I think that the biggest thing is definitely my self-esteem because when you grow up, and I guess I’m older cause I’m a mature student, but like you spend your whole life thinking that you’re not, you’re just not good enough. You’re not enough in pretty much all aspects of your life, whether it’s relationships, whether its work, whether it’s the way you look, and everything is just like, you see people, other people able to do things and you just can’t function properly and, and it’s not for the want, like the want is always there, so you know if someone is lazy and they’re just lazy for the sake of being lazy then that’s fine, but when you’re lazy, but it’s like you don’t want this, really. I’m trying to describe this, and I hope it makes sense, but like I always want the best things for myself. I strive and I have the ambition, it’s there, like I want to be successful, I want to do well, but it’s that inability to do it, and that really, really affects you when you’re trying your hardest, but you keep falling down and stumbling, and then you look back and you’re like, but I only fell down and I stumbled there because of myself because I was just being lazy. I just didn’t do the things that I needed to do. And so, then that just goes round this self-hatred wheel because you get angry with yourself for doing silly mistakes and it’s like, well if I just worked harder, if I just stayed focused, then it would have, it would have been better, and I would have done well. And it’s like that, just inability to do things is very frustrating, so you just end up in this cycle of hate. Yeah, definitely my esteem suffered the most.

##### A: That is all of my questions, did you have anything else you wanted to talk about?

L: Yeah, I guess, it’s just getting a diagnosis, and I think that in itself is super important and, because you will go through life with this idea of yourself that just isn’t measuring up, and getting the validation that a diagnosis holds is integral for anybody, I think, going through it, and, the way things are just now for people to get a diagnosis is extremely difficult and I guess that is what we are doing with our podcast, is we are trying to raise awareness and incite change in the way things are because it really does affect people’s mental health. And having to go through life and struggle the way they’re struggling when they could get the help that they need and recognize that it is just as important in a female as it is in a male you know.

#### Interview Participant #3

Interviewed by Aimee Tyrrell on 08/08/2022. Interviewee pseudonym: Georgia.

##### A: Firstly, could you tell me about your journey to receive your ADHD diagnosis?

G: And yeah, so I was like 16, and my mom sat me down because she works sort of in the learning support side of things. And she’d had a presentation at work about ADHD and she was just apparently sitting there like, oh, OK, that’s my daughter. So, she sat me down basically with, like, a list of the symptoms and sort of said, look, I think you might have this. At the time, it was ADD because I think it was like they had like ADHD and ADD separate, and she went down the list of symptoms to me and I was like ohh wow, yeah, that was me. But I hadn’t considered it then as it wasn’t really talked about.

My mum tried to get me like a referral through the GP and the GP instantly shut me down, she didn’t even see me. It was a family doctor, and she wouldn’t put it through. I was fortunate enough that I got a private assessment, and at the time they diagnosed me with ADD and offered me Ritalin, but my parents didn’t want me to take it as I was 16. So that was kind of the end of that.

And then when I was 24, I went back to college, and I realized how poor my concentration was and how much I was struggling. I was looking at everything else in my life and like I wasn’t keeping on top of things. So, I phoned up my doctor and just explained everything to her and she was like, yeah, that sounds like ADHD. And she referred me. So, I was about 11 months on the waiting list and then I was seen and diagnosed with ADHD. And I am on Ritalin now and it’s made a massive difference at college because I’ve gone from being like a failing student to like an A student, so it’s just like made all the difference for me.

##### A: Following on from that, how did you feel after receiving a diagnosis?

G: So, I went through the NHS when I went like in my adulthood, and I felt like the process was quite difficult. Like the questions they asked, and I didn’t really feel at ease during my assessment. So, like I didn’t come out of it feeling good. But then over time like once I was like on the right medication and I could see what it was like making a difference to me, that’s when I sort of started to appreciate the diagnosis and like the difference it was making.

##### A: How has having ADHD impacted experiences related to your social functioning, such as relationships or friendships?

G: I think I was quite like, I’m very impulsive, especially as a teenager. So, like I was always having like arguments with people and like could be quite aggressive and like wouldn’t think before speaking. So, I had like very up and down friendships and relationships. I was like very much on and off and like in and out of relationships and it was very unstable, which I think just sort of causes chaos around you. I think like once I was into my adulthood and like in my 20s, it started to calm down a little bit, but part of that was like finding friends who understand me and sort of appreciate it. But yeah, it was definitely turbulent when I was growing up in primary school as well. I just didn’t quite know how to maintain friendships.

Now, like, I don’t really tell people [about my diagnosis], like I tell my close friends and family, but for work, it is very much like a need to know basis because I think if you’re female, most people will just go right well you don’t look like that or you don’t have that and it’s like you can’t be bothered explaining yourself really, so there’s just no point.

##### A: Did you used to think that this behaviour was part of your personality growing up?

G: I always used to think there was something wrong with me, but I couldn’t really put my finger on it, and then like I was told that I was quite aggressive and quite confrontational and that was what was causing it. So, I would kind of question why I was like that, and I felt like it was sort of out of my control and yeah, I just couldn’t really put my finger on it. Like what was happening, and it was only like, as I have my diagnosis now, I can look back and realize like, it does affect those areas.

##### A: How would you say having ADHD has impacted your academic functioning?

G: Right. School. Bad. I skived a lot, like I was in trouble a lot. I wasn’t like a bad kid, but I was like an annoyance to the teachers, like always chatting or getting distracted or swinging on my chair. I stopped going to classes after a certain age. I really struggled with concentration. I didn’t really do my homework, like I just couldn’t apply myself and I would have to like, I feel sorry for my mum, because she would sit me at the table and she would be like, just do this past paper, like she’d have to sit me there and make me concentrate because I just couldn’t sit still. I would never sit still on a chair. I have done my work on the floor if I had it, but like, I just, yeah, could not motivate myself to do it.

I did manage to get myself back into school and get some of my Highers, but it was just an uphill struggle, and I was resitting things. When I left school, I sort of started working in hospitality and all that and then I moved into retail, and I quite liked that because it was like less academic thinking and stuff like that, and I’ve tried to go to college a couple of times, ended up dropping out. And then during the lockdown, I was like well I have always wanted to work with animals, so I was like, right, I’m gonna go and like, try get this. So, I had to go and get my school subjects, by the way, I do ramble a bit, so I hope this makes sense.

And yeah, I had to go back and get my higher biology that I didn’t get in school. And I was sitting in that class, and I was like, that was when I noticed, I was like, I can’t believe I’m still struggling like this, and my teacher noticed it as well. And my teacher was just like, I thought you didn’t care about the subject, and I was like, no, I am actually like on a waiting list for ADHD now. So, that whole year was a struggle and then I managed to get a C and get into my animal care course last year. Term one was an absolute bomb, like it was just awful. I failed. I was like, failing everything. I felt like I wasn’t like I was, I was missing questions and wasn’t answering stuff properly, and I almost dropped out. I got my diagnosis in November, and I started term 2 medicated, and like, it just went like it was night and day. I was actually like, doing quite well and passing things, and, I don’t know, it just seemed to like quiet my brain and make me concentrate better. It’s really weird, but it just makes such a difference.

##### A: Did you struggle with organization and meeting deadlines?

G: Yeah, it was like, terrible with high school work and stuff, and I think when I started working, I got better at meeting deadlines. And that, like, taught me a lot about sort of time keeping and deadlines. Time keeping is something that like I’ve been so bad at it and now I’m like the opposite, so, I’ll arrive everywhere stupid early to make sure that I’m not late. But that is also something that has definitely got better with age as well.

##### A: How has having ADHD impacted your psychological functioning? Wellbeing & emotional control, for example. . .

G: I’ve always had sort of issues with my mental health and wellbeing, like my emotions can be quite up and down. And I’ve always been quite an anxious person. So, like now finding out that they sort of like that’s common with ADHD and it coexists and stuff explains a bit more because I have noticed that like my mental state has been a lot better this year, whereas it used to be a lot more up and down. I’ve mentioned sort of like the aggressiveness and the anger and everything, I think that all just probably comes into it as well and looking back I’m like ohh right, that explains a lot as to why I had such extreme emotions.

##### A: How has your diagnosis impacted your self-esteem?

G: I was always sort of called stupid at school and like I think you sort of play into stuff, so I was like the clown of my group, and they found it funny, like I was the joke when it came to like intelligence and everything. So, I think when you think that about yourself, you don’t really make the effort to like, do better as well. I’ve sort of went through my life thinking like, oh, you’re not very smart and all that stuff, but actually, this year, I’ve been like, it’s been quite eye opening because it’s the first time I’ve been like actually I don’t feel that way anymore. Like I don’t see myself as like, less intelligent as anyone else because I have sort of got the capacity now to like focus on things that I am interested in and group my thoughts together.

I think it’s like prove them wrong kind of thing and just like seeing my difference in college and stuff as well, like seeing my grades go up, it’s like OK, so there was actually a reason that I didn’t do well in these kinds of areas.

##### A: Do you feel that there was a lack of support while you were at school/in education?

G: I think so because like I was not like the bad kid or like really good, so I sort of went under the radar a little bit. I was more like distracted if other people are doing stuff I would get involved, so I think like, unless you’re really acting out, like boys tended to do more, like you don’t really get noticed like you’re not causing an issue for them.

I was always like drawing on my book and like swinging on my chair, and teachers would be like tearing their hair out, but it wasn’t like enough for them to sort of pursue anything.

##### A: Is there anything else that you would like to talk about that you feel is important?

G: I don’t know how relevant this is, and I know the NHS is absolutely stretched at the moment, but I think further education especially on like how it presents in females because I did feel like I wasn’t really taken seriously in my assessment. Sort of like because I arrived really early to it and it kept being brought up in any question about time keeping the person assessing was like, oh, but you were on time, so that is obviously not a problem for you. That kind of knocked my confidence, so it felt hard to answer the rest of the questions. And they were like making comments like I wasn’t like fidgeting enough, sort of in their eyes, like I always fidget with my hands and stuff, but I’m not like standing up and walking around the office and that. I think those kinds of comments made me close up because you feel like you’re not being believed. So, I think there needs to be more education on being impartial and how it presents in different people, and I know like it’s not that easy.

I’m glad to help because it’s definitely something that I feel like needs more research like I can see it getting. I’m seeing more about it now, which is nice, but it definitely has a long way to go, I think.

#### Interview Participant #4

Interviewed by Aimee Tyrrell on 11/08/2022. Interviewee pseudonym: Molly.

##### A: Firstly, could you tell me a bit about yourself and your journey to receiving an ADHD diagnosis?

M: Yeah, well, my diagnosis was very late in life, in fact, it was only this year. It was probably, I’m trying to think how many years ago, three or four years ago, I can’t quite remember the exact date, that I had my autism diagnosis. Umm, prior to that I’d suffered with sort of anxiety all through my life and not perhaps been as successful as I should have been on paper. You know, I didn’t sort of fulfil my potential in many ways and struggled with a lot of issues, you know, housework, actually focusing on jobs, all sorts of things really. But the anxiety was probably the worst part for me at that particular time in my life. And I went to some, umm, CBT sessions and it was there it became evident that there was a possibility I might have ADHD and autism, so it was from there that she asked my GP to refer me on. I got the autism diagnosis first, it has taken me ages to get and for the GP to sort out my referral properly for the ADHD but got there in the end and was diagnosed with that this year.

Yeah, but yeah, general struggles all my life from childhood onwards really, with work, keeping on top of things and always being late for things. Not doing homework on time, not turning up to work on time. You know, just so many things really. But it never occurred to me earlier on in my life that was what it could have been. I just, you sort of put yourself down and think you’re just not good enough or that you’re lazy, or that, you know, just not as good as everybody else, essentially.

##### A: How did you feel when you received a diagnosis?

M: Well, much better really, because it has helped me in a lot of ways. I’m not currently medicated for ADHD, and I was considering it, but I’m having blood pressure issues at the moment, so it’s probably unlikely that I will end up being medicated, which is a shame in a way because I wanted to see what life might have possibly been like if I could get myself organized. And you know, able to do things and yeah, just to see things from the other side of the fence. However, it has changed the way I approach lots of things, having the diagnosis, so that means now that I plan things differently, and I do everything a bit differently now because I’m aware straight away, that like, this is gonna cause me problems, or this might be difficult for me to finish, or this might be difficult for me to start, so I have to sort of go about things in a different way and not beat myself up if I can’t do things. I tend to delegate more things now, so whereas I’d try and take on everything, knowing full well I wouldn’t be able to do it, and then making myself anxious and ill trying to do these things. I’m better now at sort of saying right, I’m not gonna do that because that’s just not gonna work, you know, I feel like I’m better at setting boundaries a bit now. So that’s helped me a lot, yeah, so it’s been a good experience in a way having the diagnosis with regards to my life, I can plan things, work things better. And I feel better about myself because I’ve got a reason, it’s not that I’m stupid or that I’m lazy, I know why. And it makes me feel a whole lot better about myself.

##### A: How would you say that ADHD has impacted your experiences related to your social functioning? Perhaps your friendships and relationships?

M: Yeah, definitely, I think it has affected most areas of my life, but that area in particular, yes. More so as I have got older, actually, even more so. I suppose it was bad when I was a real small child, I couldn’t, I struggled to make friends. I was so shy, what I thought was shy, and I just couldn’t, umm, deal with the whole friend business, really. I didn’t even know what a friend was, I don’t think, when I was really little. Teenage years onwards in my 20s, 30s, possibly even 40s, I created this person, I made this mask, who was very outgoing and did a lot of things. But I still struggled in intimate relationships, partners, or close friends. And I very often ended up in friendships or relationships that were a bit abusive, really, I think they took advantage of the fact that I was sort of trying to people please all the time, and made me, I think, looking back, it made me quite vulnerable. Yeah, so it’s not been a good experience in that way. Again, now I’ve had my diagnosis, things are a lot better. So, I now sort of learnt to let certain toxic relationships go, and I’ll try and nurture the ones best I can, the ones that aren’t. But I still feel, and it’s been a theme all through my life, is the rejection sensitive dysphoria, I’m very sensitive to rejection and that impacts relationships as well. And, umm, even now, with disclosure, I don’t tell many people in my personal life about my diagnosis. I am a bit more open about it at university, but I worry what some family members and close friends will think of me if I was to tell them about it.

##### A: So, I believe you’re a student at the moment, is that correct?

M: Yes, that’s right.

##### A: How has ADHD impacted your academic functioning?

M: Right, yes. So, prior to diagnosis, I’ve tried to perhaps get qualifications that I couldn’t get when I was at school because I just failed maths, I failed English. Again, I’ll put a lot of it down to I just can’t do that, that’s just too difficult for me. I’m just not good enough. And several times during the course of my life, I thought right, I need to get this done. I need to do this and know I can do more. I know I can be more; I just can’t get the paper to prove it. A few times I’ve tried to redo my English and maths and again gave up because it just felt impossible. Once I had the inkling that, yes, this is what it might be. It might be because I’m autistic. It might be because I’ve got ADHD. Then I approach, again, I approach things very differently. So, I thought, actually, yeah, I can do this. It’s not that I’m stupid, it’s just that I need to do things the right way for me. And so that’s how I started my journey going to university. So, in order to do that, I had to get my English and maths, but knowing how I am, I found the route that was easy for me. So, umm, I didn’t do GCSE English for instance, I did functional skills because that didn’t involve poetry or anything that was too imaginative. It was basic stuff, and I did the course online. I had to go in for an exam which was stressful. But the course was online so that was good. And the GCSE maths I also did online. I tried to do it in a college, but I was having panic attacks every time that I got there, I just couldn’t cope with the scenario very well. I think that this was partly a build-up of anxiety, maths anxiety in particular for all those years, it’s an awful subject if you’re not very good at it. And, and, I really struggled in that environment because it was too sensory as well. So, when [the teacher] gave us work to do, instead of leaving the room quietly, like letting you work quietly and in silence, she’d be talking over the top of the work, working time, so I couldn’t concentrate on my work. All I could hear was her voice, and then she’d finish, and I wouldn’t have started. Everybody else had finished, and then I’d have a panic attack, so I changed my tactics and then did the GCSE maths online. It was fortunate because it was during COVID so that was an option. And that worked so well for me because I was in my own environment and, I could sort of have the room set-up for me, and if I got stressed, I could just walk away from the computer, play my ukulele for 5 min, come back again, cause I need to move around as well. I find that difficult when you’re sort of stuck in a seat. I like to sort of, even though I’m not sort of running up and down, I need to feel like I’m moving a bit, otherwise I feel, it feels like I’m in a strait jacket, it’s hard to explain. So, I was able to get out of my seat, move around a bit, and I’ve got everything sort of set-up here for me, so even under the desk as I’m speaking to you, I’ve got like a foam roller on the floor, and I’ll roll my feet over that up and down. And that makes me feel like I’m moving because I’m getting the input you know, and that helps me if I have to sit somewhere for too long, I’m always rolling my feet over and over on the foam roller. So, doing the GCSE maths online, with that sort of situation that I was controlling was brilliant. If I struggled with anything, I booked a 1 to 1 with the tutor, that really helped. So, I passed both of my English and maths and then I applied to uni for the degree course, but with the foundation year as well, rather than doing an access course. I was advised to do that by an organization who help mature students, really, trying to get into higher education. And it was the best thing I did, really. I think it was much better than choosing the access course routes because I’m sort of right there in the university. By the time the degree course starts, I already know how the systems work, I will know how their platforms work, and I know the location. I know everything about the place, and I feel more comfortable and confident to start, and they’ve been really helpful. So, if I’ve had a problem, they’ve always sort of helped me out. I’ve had some equipment anyway from DSA, which again has helped, particularly the one that involves, it’s called Glean, so I can record lectures. And that’s brilliant, because I can’t always remember what they’re saying to me, and I don’t always take everything in. So, I’ll find when I’ve recorded, I can play it back if I need to, to think, ohh, what on earth did you say, I can’t remember what she said, so I can just play it back. So, it’s just been a case of getting all these systems in place to help me and sort of put me on a level pegging with everybody else, really. So, whereas they can take in the information, I can’t always, it takes me longer. Umm, with regard to studying at home, again that’s been challenging, umm, because then I’ve got to organize my time and then of course, you’ve got executive dysfunction, you know, I can be sitting here, you know, saying I need to start. But how do I start? And then I sort of, end up sort of paralysed and not doing anything at all. And I often find that during the day I’m tired because I’ve not slept very well at night because I can’t switch my brain off and I’m just constantly thinking of silly things all night. And, umm, that has an impact, sometimes what I have to do is work at night. Actually, sometimes, you know, 12 o’clock, 1 o’clock, 2 o’clock in the morning, I’m working away because that’s sometimes the only time where I feel that I can. Because I’m exhausted in the day because I haven’t slept very well the night before, so it’s been difficult, but I’ve done it, you know. So, I’ve passed the first year, passed the foundation year, I’ve learned from it, and I’ll take that forward and try and find ways to perhaps be a bit more sort of organized with my time somehow, I don’t know. I’m constantly looking on the internet for methods and ideas and ways to beat this, you know? But I’m getting there, and I feel quite happy. If I’d have done this, prior to my diagnosis, and not knowing that I’ve got ADHD, I think it would have been incredibly difficult and I don’t think I would have even got past the foundation year. Well, I know I wouldn’t really.

So, yeah, having that has really enabled me to, understand, and as I said, mainly be kind to myself really, so I don’t beat myself up if I’m struggling, if I can’t remember what they said or everybody else seems to know what’s going on, I don’t worry about it anymore. I just think, uh, let’s sort this out.

##### A: So, finally, would you say having ADHD has impacted your psychological functioning? Say, your self-esteem and general wellbeing?

M: Oh yeah, definitely. Umm, more so again before diagnosis, because I really felt myself being inferior, I suppose, to other people. I always sort of has a feeling like I wasn’t good enough. And, you know, you look around, or you go visit somebody and their house is immaculate, and everything is so tidy, they’ve got kids, and everything is so organized.

And you’re thinking how the hell do you do it? How do you do it? What is wrong with me? Why can’t I do that? Why can’t I be as organized as you? And that does leave you feeling a bit, umm, you know, like there’s something not right here, like, you know, I’m not good enough. But again, since diagnosis, I do view that very differently. And there’s a lot of useful resources online as well, that sort of help with those sorts of feelings and explain things and put things into perspective a bit. There’s a lady online, on Instagram, I can’t think what it’s called, but she’s really good. And it actually, sort of, teaches you to sort of separate, when you’re trying to keep a house, for instance, the tasks involved with that and you sort of separate that, it’s not a moral thing, it’s a, umm, I can’t put the words you see, this is another thing, you see, trying to get the words, I know what I wanna say but I can’t say it. Yeah, she sort of puts things into perspective and makes you think, oh, actually, yeah, and it does help me, you know. It does help also having supportive people around you, you know, and who are always sort of on your side and will push you forward and that’s helped. It’s not always been the case, particularly before, as I said earlier, I’d sort of be surrounded with people that I don’t actually feel did want the best for me and they were just sort of, you know, using me, really, using and abusing, but it’s not like that anymore. Umm so, moving forward, I think I feel more in control of how I feel, you know, because I can control my surroundings and my life a little bit more. I feel like I’ve got more control over things. Now I understand why, so yeah, that’s helped me. Boundaries, I’m setting more boundaries now, which I didn’t do before, I wouldn’t set any, I don’t think, and that has a massive effect on your wellbeing, I think. Definitely.

##### A: So that is all of my questions. Finally, is there anything else that you want to talk about, anything that I haven’t touched on?

M: Umm, I think we’ve covered everything really. Umm, I’m sure some people would be very, you know, feel very differently to me and being upset when they got their diagnosis, I was actually relieved, and I was very happy because finally I could put things into perspective and understand and feel much better about myself. Umm, you do spend a bit of time looking back on your life and looking back on it with a different filter on, and thinking, ohh, if only this, or if only that, but that only lasts for a brief time. And then you think, well actually really this is, it wouldn’t have made much difference and I’m quite glad things have happened now rather than earlier. I think my age, I mean, I’m 53, if I’d have been diagnosed with anything at all back in the 70s, what would have happened to me?

You know, who knows. I wouldn’t be in this position now, I don’t think. So, I’m kind of glad I had a late diagnosis, really. People understand it more now. There would have been more of a stigma back then, if you know, I don’t actually think, as a woman, we would have been noticed at all really, or wouldn’t have understand what it was. Umm so yeah, I think that’s about it really. It’s hard. It’s really difficult. Life is really hard. And sometimes I think ohhh, I wish this wasn’t such a struggle, I still feel like every day can be a struggle, and then I just have to sit back and think right, let’s make a plan and I just have to then tackle things differently, but it doesn’t stop the anxiety. It doesn’t stop, you know, having to really push to get things done, push through the barrier, it feels like you’re pushing through a barrier really. So yeah, that’s how I feel.

#### Interview Participant #5

Interviewed by Aimee Tyrrell on 12/08/2022. Interviewee pseudonym: Chloe.

##### A: Firstly, could you tell me a bit about yourself and your journey to receiving your ADHD diagnosis?

C: So, umm, I’ve always struggled with dyslexia and like, I’ve always struggled in school. It was around 21 when I got the dyslexia diagnosis, but it was only the temporary one they did at college. And then when I’d gone to uni, I needed the full dyslexia assessment that you have to go through, and I went and got that done in December, but then I was talking to someone last September, October time, and they said like I do think you have ADHD. And I was like, well no, because I thought generally like, it’s a bit portrayed by the media as well, like you think it’s just naughty kids that have too much sugar. And then I did work with a few kids and my mindset has changed from when I’ve worked with kids, but I never saw it in myself. Umm, but I went to my GP, and I know I’m very lucky because I was quickly diagnosed. Like all within 6 months, I went from submitting to GP, had the full assessment and then started medication trials and everything. I’m very lucky. And it’s like the more you learn about ADHD every day, it’s like ohh yeah, that makes sense, or it makes sense why I do things in a certain way.

##### A: How did you feel after receiving a diagnosis?

C: It was very mixed emotions, sometimes it doesn’t feel real still cause it is very new, but then my biggest annoyance was why was it not caught before and then that’s when I found it it’s more, it’s very hard to diagnose in females, let alone teenage females, and then it’s a bit of relief because things made sense. Like it made sense why I don’t process emotions sometimes, why sometimes I’m underwhelmed, or sometimes overwhelmed, why I’m just all over the place. Umm, it makes sense for pretty much everything and then it’s just a new journey of trying to find out different coping strategies and mechanisms, and just how to actually deal with it.

##### A: How would you say that having ADHD has impacted experiences related to your social functioning? Say, your friendships and relationships?

C: Relationship is sometimes hard especially, as like what seems like a small trivial thing to him, to me, I will just overthink it and everything, but we just try and talk to each like, he’s like I’m gonna say something, but I know you’re just gonna overthink it for the next few hours. Or if he said something to me, like just the other day we were doing the tip run, and I wanted to do a third tip run because I had to do it to be settled in my mind, but he was like I don’t wanna do it, but he did to please me, but even though he said to me three times, I don’t want to do it, I didn’t process it, so it’s just, we’re trying to figure out ways of me actually trying to register it, so it’s a learning curve, umm, in my relationship and with the other half, with friends, a lot of my friends work in SEN schools, or they are teachers, so they work in that kind of area, or some have ADHD themselves, and most of them thought I had ADHD but I just wasn’t’ medicated. Like they knew and they were like, we thought you knew and it’s just something you didn’t wanna tell us at that time. I’ve got a very good support system and like a lot of them knew about it, and they were like, yeah, makes sense. And then we’ve discussed things and they’ve seen the progress being on the medication and how it has helped me. My one friend, who is like not in that area, she generally knows nothing about ADHD, she’s actually taking time to do research on it as well, which I really appreciated. And it like, shows how good friends they are, because she was willing to do that. But she was like, wow, it’s like a checklist for you, it’s like yes, you do this, you do this, you do this, and in her job role as well, she’s like set up a couple of posters in the office space and that, to help with awareness of ADHD. And she’s more aware of it now and how it, like, works and stuff. Umm, so it’s kind of knowledge and it’s just we are all kind of good together.

And my mum had a bit of guilt I found. Once I was diagnosed, she was like, how did I miss it? It was like school missed it, everyone missed my dyslexia as well, so it’s like not her fault. Sometimes we can’t communicate properly, and I just end up breaking down crying because I feel like I can’t get my point across, but it’s just a learning curve with everyone.

##### A: Do you think that if you had someone, say a friend, who wasn’t as supportive as your friends are, would you hold back from telling them that you had ADHD, do you think?

C: Yes, so, you know that you have that one auntie that you can tell everything to, well I spoke to her about it and she just kind of laughed down the phone to me and I was like, oh OK. So, I was put off going to the doctor’s for like a month, but then I thought, you know what, I needed answers. And then when we said I was going through this to get the diagnosis, she just kept really quiet of it. And when I had the official diagnosis, like when I was diagnosed, my mum told one of her other sister’s and she was like oh it makes sense and since I’ve got a bit closer to that auntie. I’ve found that we’ve been able to connect and bond, she’s more aware and that, she’s like, remember when you did this, it was your ADHD, that’s you, and I can talk to her more openly about it. And my other auntie is just, like really supportive, and she’s like, oh you’re just crazy darling, and that we love you. So, it’s gone from the auntie, who you can tell everything to, to laughing down the phone, saying it’s all made up and it’s all lies, and saying that like she knows people with ADHD, and I haven’t got it. And she was very unsupportive, and I was really shocked as well, I was hurt, so our relationship has really changed, like I don’t talk to her as much anymore, and partly because I’m like what’s the point in talking to her when she doesn’t understand what is the one thing that actually makes sense in my life, but it is what it is. I’m a great believer that everything happens for a reason. My other two aunties, which I wasn’t as close to before, I’ve kind of gained a better relationship with them, and I still talk to the other auntie about other stuff, but I just don’t bring up the ADHD. Like, she says she knows a lot of kids with ADHD and autism, and it is brought on by their diet and that makes them crazy when their parents give them processed food and, so yeah, she’s a bit like that. Like, she’s obsessed with what the media teller her and Facebook and conspiracy theories and stuff like that.

##### A: As a student, how has ADHD impacted the academic side of your life?

C: I did my first year at university, I did full time because it was 2020 and I had nothing else to do. And I was going through the dyslexia kind of process at this point, and I had one tutor who was very supportive, and I had another one who didn’t believe in dyslexia, ADHD, autism, and everything. She just believes it was a lazy excuse for not going to school. And then I was supposed to have her for second year because of exams, and I know I don’t do well on exams. I thought I’d relieve the stress and I’d just do it part time, which I have found has been easier to cope with, especially with time management and organizing, and I can just focus on like the one thing instead of having to jump between things. I was trialling on Concerta ADHD medication, and it was very, it did not work for me. So, I found that uni did get a bit hard at one point. I was supposed to have that old, nasty tutor, but I just put in a complaint and said I’m not having her, please remove me, so now I’ve got a really lovely tutor, who was supportive. She even sent me like ADHD links as well sometimes. And, I had an extension, like 2 times, and she was like you’re not submitting anything, I’ll give you a grace period, but she was like, just try and get something done and everything, and it really worked. I found just doing it part time I was able to cope better with the ADHD and focusing on what I was learning, and it was easier. And then I had like tutoring sessions as well. I had a couple sessions from the tutor where we just went through what dyslexia was and what ADHD was and like figured out different coping strategies and mechanisms I could do to help with uni work as well, and I’ve got that tutor until I graduate now. Umm, but if I have any question about ADHD from my tutor, or from the disability support team, they will always provide information and kind of help me the best they can as well.

##### A: How would you say having ADHD has impacted your psychological functioning?

C: I think, I think at the minute with my psychological wellbeing, it’s like trying to find the right medication for me and the right doses. And so, I’ve been doing it since March and then Concerta didn’t work, so then I had to go through withdrawal for like a month.

Withdrawal was worse with the side effects than I had on the actual medication, so I felt like I was getting a bit low sometimes. With Concerta, I was emotions everywhere, like I could drop a pen, and I would cry for like 20 min. I was very emotional. Like my other half would have been looking at me like, what can I do for you? And then I dropped a plate, and you would have thought I’d just killed someone with how hysterically crying I was. I found that with the medication and then my doctor was like, oh, this might not be the medication. Then one of my friends wrote me an email saying there was like a one in a million chance that I have this side effect, which was what I had. And even my friends noticed that I wasn’t normal [Chloe], I was very just like “How are you doing”? **Blunt tone**. Normally, when I jump in the car, I always have like a story or gossip to tell, but they said to me like something is not right with you. I felt like, I was like, I felt physically fine, I knew I was a bit washed out, but I thought maybe I’m still trying to process the diagnosis, umm, and like with uni and everything and worrying about exams. But so, my friend has got a toddler, and even he was like, didn’t recognize me because it wasn’t the same energy I usually had. I’m on Elvanse now and that seems to do a lot better. And it’s amazing who you speak to about it, and they’re like, this didn’t work for me, or this worked for me, and you feel like you’re not alone on some of the groups and some people you talk to. We do have bad days, but we do have really good days as well and it’s just finding the right balance. Finding and having that support system where you can talk to people. I do find it is getting better, but if I am upset, I try and write something down. I try and figure out what might be causing it. If there are no obvious reasons why I might be upset, I’m like, OK, maybe it’s just my ADHD brain and maybe it’s something that I’m just not seeing and stuff. I have said to my friends and family, like, if I said something that I might not realize might hurt someone and that lot, or like, if you’ve said something to me three times, and it’s still not processing, like we will try to find a way that it will process in my brain. And like the only way I’m going to learn is if you guys tell me and teach me. And so that’s the new strategy we’ve been doing like the past few weeks as well.

##### A: Would you say that since having your diagnosis, you understand yourself better?

C: Yes, so, before, people would be like “oh, that’s just [Chloe],” and we still say that now, but we know it’s the ADHD behind it. Like, a little like, I wouldn’t realize that I would interrupt people during conversations, like, sometimes for me it’s like I’m gonna forget what I’m gonna say, so like that sometimes I didn’t realize. And then once I was diagnosed, I would be like oh do I do this? And they all look at me like yes, so if I do do it, I’m like please tell me so I can learn. I followed this page online, and it just tells you like different things for ADHD and stuff and there was one about showering, and like sometimes I didn’t want to shower for a few days when I was a teenager and when I was younger as well, and we couldn’t seem to figure out why. And then like, once we read about it and we done some more research on it, I was like yeah that makes sense why that happened, because of sensory overload.

Also, I used to be pen pals with my cousin and I kind of wanted to stop so I sent her all the letters back, and she cried. I didn’t see, I never saw that I did something wrong. And later on, we were talking about it, and she was like I don’t think you realize how badly I was hurt over that, I just thought that’s what you do, send all the letters back. And now looking back at things, it makes sense why I did that. That would be the ADHD and my brain.

During 2020, once the borders opened, I was in France within 4 hr. Told my mum I was going to France, and then she phoned me the next day like where are you, and I was like I’m in France. Sometimes I have compulsive spending, like I spent like 800 pounds in like 2 days back in October last year, and then we kind of realized then I returned the stuff, like I didn’t want the stuff, I didn’t need it. But then sometimes I would tear up the receipts so I couldn’t return things anyway, or I forget where I’ve put the receipts. But yeah, like, it makes sense of why things like that happen and stuff, but it’s just like, little coping strategies for things.

##### A: Finally, is there anything else you would like to add?

C: I think we have covered everything. I know I am very lucky with the support system I have and how I was quickly diagnosed, and I have found good tutors, good friends, and that lot. But I know some people don’t have that. And yeah, but I think as well if you go in with more of an open mind and think like, OK, this is the cards you have been dealt with in life, you can either go one way and you can control your life, or we can find ways to like to try and improve and learn and understand more about yourself.

#### Interview Participant #6

Interviewed by Aimee Tyrrell on 12/08/2022. Interviewee pseudonym: Hannah.

##### A: Could you tell me a bit about yourself and your journey to receiving an ADHD diagnosis

H: Uhh, ohhh, I was diagnosed after my first child. And when I told my parents, they said it was mentioned at school, but they never bothered following up with it because they didn’t think there was any point to it, because I already had a dyslexia diagnosis. I’ve got, uhh, an undergraduate and just starting a masters as well. And I’ve done several jobs, I don’t work in my degree area anymore, I did for a little bit then it got a bit samey and a bit boring. So, I now work [in healthcare].

##### A: What led to you seeking a diagnosis?

H: Umm, I’ve got a friend who’s got ADHD and she noticed a lot of similarities between us, and I’ve also got a friend who is a clinical psychiatrist who said it seems like something I should probably follow up with, because I started, I sort of had a suspicion for a lot longer, but didn’t bother following it up because it wasn’t, it seemed like a lot of work, actually, and it wasn’t impacting my life enough that I felt like I needed lots of extra help. I had my own coping mechanisms, but then after I had my first child, it was sort of, lockdown was imminently after sort of thing, and it was, it got a little bit overwhelming. So, my friend that I was speaking to about it, who had a diagnosis and was on medication, said that, you know, it really helped her, and it was worth going for it. So, I did. Going from my initial assessment to being treated, it was probably 3 months, which I think is pretty epic compared to some of the wait times that people are facing at the moment. Uh, and I found medication really, really helpful.

But at the time when I was struggling, I was on maternity leave, but still completing a qualification with work that I was doing, so I should have been using some of the maternity leave to catch up on work that I hadn’t done when I should have done. And I didn’t, and I just found it all a bit overwhelming, and I couldn’t deal with the distraction of having like a kid and more plates to spin. Finding the time to sit and do it became harder, which meant I couldn’t do the, you know, leave it until the deadline makes you do it kind of thing, because inevitably you’d have a child that didn’t sleep of suddenly got ill or something like that. And so, it got to the point that was pretty bad and I got referred to the education department at work for additional support. And I sort of thought to myself that, umm, I’m going to get in trouble with it. I need to sort it out, this sort of thing. So that’s really what prompted it because it was starting to impact work and home life and getting things done. And I didn’t want it to get worse and get to a point where I did something stupid looking after my daughter or forgot to take her to a doctor’s appointment and things like that. So, when it was just impacting my life a bit, it was one thing, but when it was like the prospects of impacting my child, I was a bit like, OK, need to see this and but then after the diagnosis I didn’t realize how much of the stuff that I’ve struggled with. Like my whole life was ADHD, I just sort of assumed it was my personality. Well, I guess it is, at what point is a diagnosis a personality, I don’t know, but, yeah, so, it was good.

##### A: How did you feel after receiving a diagnosis of ADHD?

H: If I’m really honest, I didn’t really feel anything. I think a couple of people, including my mum, thought I was going to be really excited about it. To like finally have answers and stuff, but I was more looking forward to the fact that now there was a formal diagnosis, there was like some stuff I could try to see if it helped. Like, I don’t actually have any particular feelings about the diagnosis, like, I don’t think it’s changed the way I view myself other than maybe a bit more self-compassion. But the diagnosis itself, I was just a bit like, OK, like, not really excited, but not really – like I’ve seen some people when they post stuff online and it’s just about how their world has shaken, now they’ve got this diagnosis, and that wasn’t really how it was for me. It was just, OK, cool.

##### A: How has having ADHD impacted experiences related to your social functioning?

H: I think it’s always affected my friendships, because I have had several nasty fallings out with friends and stuff, and the general theme when it happens is that I can be a little bit much sometimes and they weren’t nice about it. And as I’ve got older, I think people are just a bit more understanding about differences between people, whether or not you’ve got a diagnosis or anything. But I always find it quite difficult to have like, friendships, and to hold onto friendship groups. I have lots of friends and like mates, but not like a close core friendship group that some people have. So, I’ve got a couple of good friends that sort of dip in and out, but if I’m honest, I think all of them are sort of neurodiverse, and we are just a bit more understanding of each other, I think, but, I don’t have trouble being social or being in social situations other than when they get a bit overwhelming and it all becomes like, like a really long extended day with lots of new people and stuff, I’d probably rather go home at that point. It’s not an environment that I enjoy, but I can cope with it when I need to.

##### A: How has ADHD impacted your academic functioning?

H: Umm, it probably made it much more stressful, because of the tendency to leave everything to the last minute or on one occasion restart a piece of work that was worth most of the module the night before it was due, because I decided it wasn’t good enough and changed my mind about what I wanted to write about, and so, I think, probably if I had better ability to forward plan and well, not even planning, I’m pretty good at planning, but just following through with the plan is the tough bit and it probably would have been a bit less stressful. I can be a bit on the impulsive side, so like for my dissertation for my undergrad, I bit off a bit, probably a little bit more than was appropriate because at the time of submitting the proposal I was full of enthusiasm and, umm, then it got quite stressful and quite a lot because I took on quite a lot. I mean, there was a couple of stupid things like I missed out on a scholarship in my first year because I forgot to set my morning alarm and slept and missed the exam. And the exam was only 5%, and I needed 90% in the module, and I already had 90% so I could have gone to the exam and not actually written anything, and I’d still have got the amount I needed, but the fact that I didn’t attend the exam meant I was not qualified for the scholarship, so I missed out on the scholarship that way. There was once a time where I went on a night out the night before a 70% exam on one of my modules as well, which was really stupid and I was extremely unwell the next day, during the exam probably. Again, was not the most sensible of things. I mean, luckily, it was the subject that comes quite naturally to me, and I quite enjoy, so actually, I managed alright in the exam. It didn’t mean like, I didn’t fail my degree or anything because I’m a bit impulsive, but definitely, in some aspects I probably haven’t done as well as I could have done in terms of grades on specific modules, and on the modules that are a bit boring. You know, the ones that you just have to do because they’re the core modules within your degree, but like then you don’t really wanna pay much attention to them. There was a really marked difference between the grades I got in the choice modules and the ones that I felt like I had to study or didn’t enjoy. Like, my average grade in the modules I liked was about 85% overall per module, and the ones for the compulsory study or the ones I didn’t like was probably about 60%, so I still did OK, but it was quite a marked difference between the two.

##### A: Do you feel that you’ve had enough support for your ADHD throughout education?

H; Umm, no, probably not. I feel like a lot of it is lip service. In that you get a lot of, ohh, yes, you’ve got ADHD, dyslexia, or whatever. It’s something that you need support with. And then at the beginning of the time you get a lot of, how can we support you? What would help? Ohh, you want to have notes in all of them or all this sort of stuff or having recordings of the lectures helps you, OK great. And then actually when it comes to putting those in place, it’s inconvenient and it kind of falls by the wayside. Then, then were a couple of times where I ended up getting in trouble, well not like I was doing something I shouldn’t have done, but I was finding it really challenging because the things I’d asked to be put in place weren’t put in place. And then I’d been told I could record lectures, voice record lectures to redo notes, and then I’d go to voice record them, and the lecturer would say no, even though they had said I could. And stuff like that, and some lecturers were really great, and some teachers as well, if you think all the way back to school, you get some people that sort of try really hard to like help or support you and then you get some that sort of go, ohh yes, well you have ADHD but it doesn’t mean that you can’t study hard and like, yeah OK, thanks. They just don’t understand, I still think in education, there are still a lot of teachers who, I think they think maybe they think they understand it, so they don’t read more about it because they think they know what it is, but there’s a lot of the, umm, perspective, misconception with sort of ADHD being only hyperactive teenage boys, and oh, you can’t have ADHD because you managed to get a degree, or you can’t have ADHD because you got A’s in those exams, you can’t have ADHD, for all sorts of stupid reasons. And oh, you haven’t been fired from every job you’ve ever had, you can’t possibly have ADHD, sort of, yeah, a lot of that sort of stuff, and obviously it is a little bit more in depth than that. But, yeah, I think there is still a sort of point of view that if you’re not completely burning out and completely having a meltdown about absolutely everything, then you’re not struggling, or you don’t need extra support. Which is obviously not true.

##### A: How has ADHD impacted your psychological functioning? For example, your self- esteem?

H: I think that is probably the biggest effect it has had on me. Yeah, I think ADHD has had a massive effect psychologically. More specifically because not being diagnosed younger has had a massive impact on me. I was being told things like “I don’t know how you will cope in the real world” and that I must not care about things if I lost or forgot about something, it made me feel like there was something wrong with me and that other people could do things that I found really challenging or kept messing up. Umm, I found I believed that people had to put up with me rather than enjoying my company and I had to apologize for things a lot that I shouldn’t have and didn’t need to.

The impulsive side also means that I’ve struggled a lot with my weight a lot, due to bad eating habits and comfort eating. I have also always exercised a lot but have found that I hate the gym because it is so boring and so I need fun sports and activities. The issue is when I fall out of the routine or get bored of one activity, I find it difficult to start something else or get back into routines I had. So, uhh, yeah, I haven’t always had good body image, I have often been called weird and that I wasn’t meeting my potential which led to low self- esteem. Since a diagnosis, my emotions have become a lot easier to handle, both after being medicated and because I understand them more so give myself more grace. I get anxious a lot too because I am more likely to have forgotten something or mess up or get lost or run late whatever when going to new places or doing new things which means I find it hard to socialize outside of my comfort zone.

#### Interview Participant #7

Interviewed by Aimee Tyrrell on 18/08/2022. Interviewee pseudonym: Jessica.

##### A: Could you please tell me a bit about yourself and your journey to receiving an ADHD diagnosis?

J: Basically, I always thought I had a few quirks and stuff but like I majorly struggled in school, but I thought it was just, uh, you know, I still performed quite well and still got good grades and stuff, and so, they told me I’ve got dyslexia in my first year of uni. This was like when I first went to uni like, when I was like 22 or something. And I thought that answered a lot of questions, but then my second degree, which was in like an NHS environment, I was under quite high supervision and like my supervisors were picking up traits and stuff which I had. Initially, it was just a few traits, a few little things, and that. On my first placement like I used to like to click my pen and stuff when my supervisor was talking, not really realizing and she would obviously be a bit negative towards that behaviour because it might convey like, you know, not listening or whatever. And then after a few weeks, she was like do you need to do that like is that something that you need to do to be able to concentrate? And then as I was kind of like piecing it together, I was kind of like, well this is very like symptomatic of not being able to listen when, you know, there is lots of information. So, I thought you know that might be likely to be linked to dyslexia, but then as I did a bit more research and stuff, more behaviours were being picked up. It was things like, interrupting someone, like when my lecturers were speaking for example, like obviously that might seem really disrespectful. I would like try to paraphrase what lecturers were saying because what they were saying was too long for me to keep concentration on. People were making comments and I was getting into trouble for things that I realized were symptoms of ADHD. I felt that it was like unfair because they were picking up these things which related to symptoms of ADHD, and I’d said that I was going through the process to get an ADHD diagnosis. It was kind of messing with my head a little bit that, I was thinking that this is discrimination arising from disability, you know, in terms of these traits or symptoms whatever were coming across as really annoying to the people I was working with, you know, like in terms of focus and stuff like that. I wasn’t trying to be disrespectful, but that is kind of how it felt it was. And they didn’t implement my support plan, like there was various different failings. I had like one supervisor, who was like, you need to focus on this, it’s really important, but I was just thinking like this is so overwhelming, there is a lot going on, there’s lots of notes to make, lots of things going on in the wards, and I just found it really difficult to focus in that environment. And then I think what exacerbated it was not knowing how to kind of manage it or what was going on. So, I went to get an ADHD diagnosis and a psychiatrist diagnosed me with ADHD and confirmed that I had ADHD all along. This was about 4 weeks ago now. I felt constantly like attacked and like discriminated against, so obviously like that impacted my interpersonal relationships at work, and in the end, I said, look, I feel like because of the comments you’re making, like I feel this is discrimination. And then I was pulled off that placement because it was impacting my mental health and it was impacting the team, like they could see I was suffering.

##### A: How did you feel after receiving the diagnosis?

J: It definitely made sense, it does make sense and I’m probably a lot more compassionate to myself now, I think instead of like taking on too many things at once, and thinking yeah, I can cope with it, but I think that exacerbates ADHD because like when you’re stressed, I think the symptoms are more prevalent. And maybe, I think because of ADHD, I think you’re inclined to do lots of different things, uh, and it burns you out. So, I guess like in knowing that I have ADHD and I felt like I’d been running on a motor for like, however long, always busy, and always doing things, umm I guess it’s kind of made me stop and just go, right, just work one job, just do one thing. You know, get things sorted and like figure out strategies that are related to ADHD so that you can better manage it. It’s kind of made me think, right, well, I can’t function how I have been functioning because it’s not good for me or maybe anybody else.

##### A: How has having ADHD impacted experiences related to your social functioning?

J: Yeah, it’s quite bad actually, because like, although I’m quite a sociable person and whatever, sometimes I’ll get too busy with like studying and work and like, I kind of neglect personal relationships at times, because I’m so busy doing whatever I’m doing. A lot of the time I’ll have lots of associates, but then few people that actually know me like very well.

Umm, and I think it’s because my focus is just so bad, and I’m so interested in lots of different people and lots of different things, it’s difficult to just stick to one group of friends. And I think it’s made romantic relationships really difficult because, you know, like things that I struggle with on a day-to-day basis, which I thought and think are normal, like probably aren’t, so it takes me a lot more focus to do simple things like organizational things and like when I’m doing those organizational basic things, like I don’t know, thinking what I need to take to work, it makes my brain work a lot harder. So, I think in the moments when a partner was around, for example, I could get quite annoyed by the fact that like they were even talking to me whilst I was trying to do something like that, which obviously doesn’t create a nice environment. I’d explain that like I can’t really do two things at once, like I just need to get my stuff together, but yeah that’s difficult and that puts strain on a relationship and stuff. I think as well what is really overlooked is some of the emotional dysregulation that you have, you know, when things are a bit more stressful, I think that’s where I’ve been like more susceptible to snap. And like, feeling those emotions quite intensely, and I feel like that is hard for anybody to understand too. Like I can’t function whilst you’re talking to me, which is really hard.

##### A: How would you say ADHD has impacted your academic functioning?

J: Yeah, I mean, to be honest, I can kind of cope with the academic side of things, things like assignment and stuff I tend to get quite good grades. I’d have trouble with like understanding what the assignment is and understanding exactly what they are asking for if you know what I mean. So, I guess without things being clear, whether that’s the assignment brief or whatever, like I struggle to get it on the page in like an order and understand what I need to do. And like you could argue that is a very basic thing that a student should have, and they weren’t always very helpful in explaining things to me either as you know, that was what they were trying to assess in the assignment, but it is something that I also need extra help with to get me on a level playing field. I also struggled on placement with being misunderstood, like I felt that people misunderstood my character quite a lot. Like, sometimes I would be super stressed and like hyperfocusing on things, but I think to some people that comes across quite hostile, like not wanting to chat to people whilst I’m working and stuff. Which I found really overwhelming.

##### A: Finally, how has ADHD impacted your psychological functioning?

J: Yeah, like it has really affected me. I think it affected me when I was younger a lot more. I think in school because I think everybody gets affected in school anyway, but like, I think it affected me more because it was like, so up and down like a bit hyper and dysregulated.

And I wanted to be everyone’s friend and then not understanding the social norms that you can’t be everybody’s friend. Like we have cliques in school and people don’t do that, so I think I just didn’t understand that I was thinking well, like why can’t I be everyone’s friend? I thought that was normal. But like in school, I think people are like, well, no, this is our clique, don’t impose or whatever. And then I think it led to a bit of like you know self- sabotage and self-esteem stuff. Then I think when I got to like a bit more of the stuff that I am interested in, I think that kind of just disappeared because I was like well, I don’t really care about that. I think you become a little bit like well, this is actually who I am and like I don’t really need to change for you. And if I’m not a bad person, like if you don’t like that, then that’s totally OK. Like you just become a little like that. But then I think in a medical setting, like on my placement, you can’t just say, well, if you don’t like me, that’s fine, because that isn’t very professional. And I think that’s why I struggled more on my placement. But yeah, I think like recently it’s definitely taken a toll on me just because, like, I felt like my character has been like, in question. I’m like, yeah, I kinda get what you’re saying, perhaps it’s not communicated very well, but yeah I didn’t feel like my best self in that environment and that’s why I didn’t finish that placement.

##### A: Is there anything else you wish to discuss? As I said earlier, I am looking at ADHD in females because it is quite underrepresented. So, is there anything else you would like to discuss relevant to that topic?

J: Well, like everything, early diagnosis is better, because then you would have the coping strategies to be able to kind of avoid these situations that kind of lead to miscommunication or whatever. Like I think, looking back to the start of my placement, if I could have said like I have ADHD, this is how it affects me, things would have probably gone a lot better. Like, you know, if I’m coming across like this it just means that I’m focusing on my work and it has nothing to do with you, so please don’t take it that way. So, yeah, if I had my diagnosis and could have gone about things like that, things would have gone a lot better, so yeah, early diagnosis is important.

#### Interview Participant #8

Interviewed by Aimee Tyrrell on 18/08/2022. Interviewee pseudonym: Grace.

##### A: Firstly, could you tell me a bit about yourself and your journey to receiving an ADHD diagnosis?

G: So, I’ve kind of always known that I was, I was very active as a child. My mum worked in education, so she had always kind of known, but I think because it’s like, I don’t know, the different like maybe stigmas, like there’s a lot of different reasons why we kind of didn’t get a diagnosis as a child. And then when I moved away to university for my undergrad degree, I really, really struggled. I struggled in school a lot, but when I moved away from my mum, I was like, oh my goodness, this is very hard. I still kind of didn’t really understand why, because I think my perception of ADHD was just being very active. And I kind of didn’t connect everything else, and then kind of plodded on through uni and then I got a job as a 1-to-1 in, umm, an infant school, and it was the SENCO, that uh, said to me, I’d like, lost my gym kit that day or something and I’d come into school and I was just like, oh my gosh, I was just absolutely have a kind of, one of those things, like, another thing that I’ve lost. And then she kind of said, have you ever thought of, you know, that might be this? And then she kind of showed me this sheet of like more girl characteristics and it was just like, tick, tick, tick. I was like, I didn’t realize that this was all of this as well. And then I went to the GP, who was not kind. I was very, I was very overwhelmed because obviously it’s taken a lot to go, so I kind of started crying and he was just very dismissive and said ohh like I think you’ve got, I think you’re depressed. And I was like, I know I’m not depressed. I kind of said, well can you refer me anyway, and he did. So, I was on the NHS waiting list. And then the following year, I started my training at university, I had kind of struggled a lot in first year, but it was that kind of realization like OK like I kind of know why now. And then it was lockdown, and I lost my dad at the same time as well, so it was this kind of like all-encompassing like ohh my gosh, I’m really, really struggling working from home, and because I’d seen quite a lot about the right to choose and stuff like that, so in the end I thought, I’ve not heard anything from the NHS for about two years, so this was last year, like February last year. So, through the right to choose, then it was last August I got my diagnosis and then I, interestingly, got a letter from the NHS because I’d moved out of the area so that knocked me off the waiting list again so, so yeah, that is kind of the long version of how a diagnosis came about, yeah.

##### A: How did you feel after receiving the diagnosis?

G: Umm, kind of like, very relieved, very validated, I think. Because I kind of knew from like the conversations I’d always had like with my mum as a child and then her working with children with ADHD, and I kind of, I’d kind of known, but then I always, always had that, like, what if I’m just being dramatic. Like, what if it’s all in my head? So, it was a massive sense of relief to be like ohh my gosh, like OK, since someone else has kind of confirmed it. Which was yeah, it was really validating, and then, then it’s probably been like a year on and I’m kind of going through like different stages now. So, but yeah, I think relief would probably be the key word I think, yeah.

##### A: How would you say ADHD has impacted experiences related to your social functioning? For example, your friendships and relationships?

G: Umm, I think, I’ve always kind of been quite privileged in that, like my friend’s always kind of protected me and looked after me a lot, and I think I’ve always been, I mean in primary school I was bullied, but in secondary school I kind of made strong relationships and I was kind of very well accepted there. But I think that the negative side of it was, and it still happens, is that I just find it very difficult to reply to people and like following through with plans, and that kind of not being present in conversations a lot or just talking about what I want to talk about, or like dotting from different things that I want to talk about. But that, something that my friends have always liked about me is that every kind of story that I tell is like, it’s like a movie or something, which is nice. But then I think when you become aware that you do that and that it is kind of not considered to be typical, I guess you get kind of a bit paranoid about it, so I think in terms of like meeting new friends, it’s, I think definitely as an adult that has been really difficult. Like trying to pick out which things you want people to see, if you get me, I’m not too sure. And one of the biggest things is that kind of sensitivity, I was always very, very sensitive and like that perception of oh they’ve not texted me back they must absolutely hate me. Umm, I think part of that as well is then not being assertive with confrontation, that’s something I really struggle with because of that, like fear of rejection and things that I’ve definitely struggled with and that I still struggle with it now. It’s like when I moved to university, and my friends had like a different group chat, and it was literally because I wasn’t there and I never replied anyway, but like that crushed me for years and I got really, really upset over it, but kind of knowing about it, I think that’s something that I would have, I guess, what’s the word, sorry, I get really bad brain fog. I think I would have benefited knowing as a teenager, about that kind of emotional side and that friendship side of like why I’m taking things so personally and I think the impact that has on relationships has been pretty difficult.

##### A: How has ADHD impacted your academic functioning?

G: Umm, again, I think I am aware of the privileges that I had with my mum doing her job, my dad was academic and that as well, so I’ve always had the impact of having professional parents and, this is something that I’m kind of unpicking, and how it impacted what and how I achieve. I was very lucky that my mum had enough awareness and patience to get me through school. I always, always needed a lot of support from her. In primary school like, I was kept in a lot because I just didn’t do any work because if I wasn’t sat next to someone that I could copy off, and it wasn’t that I didn’t, kind of, I did know what I was doing, but I also like, I didn’t know what I was doing, so I kind of needed someone to kind of come and remind me and talk me through it again, and I found that really difficult. I found teacher relationships very difficult because of that rejection side. At secondary, like as always, the things I’m interested in, I’ve always excelled, and the things that don’t interest me, like, just, I’ve not done bad at, but it’s been very hard to kind of motivate myself to do it and kind of make those connections. I think at university, I’m always kind of like a bit leave it to the last minute or close to deadlines and things like that. But for like things to click it takes me a long time to kind of process and apply them. So, I think with university, in my undergrad degree for two years, I kind of was like ohhh like it’s just a big party like it’s fine, and I think because a lot of the time I could just wing it almost, in certain things, I would just do that. Again, my mum gave me a lot of support which I didn’t realize at the time, but like things like proofreading, I find proofreading and like those like little end bit things so difficult because by the end of it, I’m just a complete burnout, and then during my undergrad, my mum was like, I’ll proofread it for you, and I kind of didn’t realize the extent of how much I needed that. Maths wise and things I’ve always had a very specific memory of my maths teacher in Year 11, like actually screaming over me because I just didn’t get something, I couldn’t kind of, I just couldn’t hold it in my head. And again, kind of not knowing why was just so upsetting and I can still remember it to this day, I mean me and my friend laugh about it now, but at the time it was awful. And thinking back, I know it’s because of all these reasons, and if I’d have known, I would have been able to explain. Maths I always found really difficult, I still find it difficult now, but being an adult, I don’t need to know, well, you know, I can google things. I think with my studying now, it’s, I’m finding that there are similar patterns of what I am finding difficult, like understanding what was specifically meant and how to kind of break it down. I’m finding prioritizing things and time management very difficult. Like concepts and reflecting on things I am really good at, I really excel at that, but then it’s kind of something I’ve always struggled with is writing and kind of, like making things actually make sense, if that makes sense. Like all that organizing my thoughts and then combining them almost and linking them, that’s what I’ve always struggled with and I think, it’s just been very frustrating because, I’m, I have all these ideas, I know what to do, I know what I need to write about, but I just can’t do it, because it’s so much work and there’s so much of it, it’s very all- consuming and I think that’s ultimately what led to me getting a diagnosis. Because I’ve just returned to studying, I was off for a year, I got COVID and then I had to take time off and come back, but I’ve also recently started medication, so that’s been very interesting to kind of look at. Well, my attention and like concentration is like hideous, uh, yeah, and I think throughout school, again I didn’t realize that I was kind of very physically hyperactive as well, so that was trying to kind of squash that up and trying to pay attention to everything and that was hard. Umm, but then just constantly talking and having those messages of like if you applied yourself then, you know, you’d be really clever, and the kind of impact of that and then since starting medication, it’s been, it’s been quite life changing. Like now I would say 9 times out of 10 I can kind of sit down and do my work.

One of the biggest struggles I had before was, especially with uni, was needing to sit down every day and do work, it was like, how do I force myself to do this? And I was kind of just waiting for it to kick in and then running with it and then doing like ridiculous hours because I’m trying not to like lose concentration and make the most of it, so that’s been a really big thing, that kind of consistent focus. I think another really big thing that I’ve noticed is being able to, I guess like being able to make sense of things. Like I can read things and it doesn’t take me 4 times to understand them, I can just read them and understand what it means. And it’s yeah, there’s a few different little things that I kind of never realized how much it was impacting me until they’d been squashed. Yeah, just trying to make connections between things, I’ve kind of always described it as having one thing here and one thing there, but I’ve needed like something in the middle to help connect the two, and like medication has kind of acted as that to join it, which has been incredible. So, yeah, that’s probably a very long extended answer.

##### A: How has ADHD impacted your psychological functioning?

G: Umm, I think, as a teenager, I really, really struggled. I had eating disorders, I struggled with self-harm, because there was just so much emotion and I didn’t know why there was so much emotion, and I didn’t know what to do with it. So, it was really difficult to, kind of, process it all and then I think something that I’ve realized is that I’m still realizing, like when I got a diagnosis that you know not everybody feels like this and it’s that kind of looking at other people and thinking well why can they do it, and why can’t I do it even though I’m trying so hard. And I think that was a really big thing for my self-esteem and kind of wellbeing, like, why can’t I do it? Why is no one, kind of, almost like believing me that I can’t do it? Again, I have been very lucky with the support my parents gave me because they’ve always believed and kind of pushed me to, like say, we know you can do it. And I’ve always been really, grateful of that, but I think the kind of impact the teachers had and kind of not believing in me, and the impact of relationships on, kind of, like not being perceived as typical, that has its toll on your wellbeing. And you think, well why am I not?

And I think it is, I’m starting to kind of go through since my diagnosis, is opening up a lot of different cans of trying to self-heal really. I think different things kind of crop up and it’s, I think I’m not sad for not having a diagnosis early or anything like that, I wouldn’t change that, but it’s kind of just feeling really sad for that little person that didn’t understand and they kind of took it out on herself because nobody realized how bad it was. Even as an adult, like once I’ve learned about emotional regulation and things like that, it’s been so helpful for my wellbeing, like learning about rejection sensitive dysphoria. And umm, I’ve got quite a strong sense of justice and realizing things like that has been helpful so I can kind of give myself a reason now as an adult and understanding that has really impacted me. And that kind of dismissal from professionals and the number of times I’ve been told that I’m depressed, it’s kind of like, well, no I’m not, but like I get why you’re saying that because I am very emotional, and I have those kinds of highs and lows of emotion and that does impact your wellbeing. I think, yeah, throughout school as well it is that self- esteem, like with maths, I was just like, clearly I can’t do it, because I was trying and I was listening, but I just can’t do it, and it’s kind of what you carry throughout. But I also know on the other side that I am very privileged and perhaps my parents would not settle because they knew that I could do it, it’s just kind of that impact of different teachers and I think I guess like the strain of the curriculum and things like that. Like it just wasn’t fit for me, and it’s something that I am still battling with at university now, like I can’t’ demonstrate my strengths, and yeah, it’s quite tough knowing all this but then I can’t change anything, so it’s thinking about how I can move forward and that is something I am kind of trying to battle with at the moment with my wellbeing. But yeah, since being diagnosed it has been really incredible, I’m very grateful for having a diagnosis, but it’s just kind of unpicking of years and years of feeling not great about yourself and, yeah, kind of not knowing why.

##### A: Is there anything else that you wish to mention?

G: The only other thing I did remember, was, umm, kind of how it did impact my learning and something that I am finding at the moment with medication is that kind of hyperfocusing, and like the difficulty in breaking away from that or trying to use it and the impact of that on relationships and friendships and work as well. And I think sometimes that has definitely impacted like, not answering the question and I’ll write about what I find interesting but it’s not actually specific to the question and then going down a rabbit hole and spending hours and hours, and yeah that kind of time blindness. Like trying to do the same amount as other people, and they might take an hour to do something but instead it has taken me a whole day and I think that is something that I still, really struggled with, like kind of anticipating how long things are going to take, and again, I guess that kind of self- esteem of have I done it right? Are people going to be disappointed in me if I haven’t done it right? Well, I’m gonna have to focus on it for another five hours to make sure that I’ve done it right.
